# Mapping the composite nature of clay matrix in mudstones: integrated micromechanics profiling by high-throughput nanoindentation and data analysis

**DOI:** 10.1007/s40948-024-00864-9

**Published:** 2024-08-14

**Authors:** Xiangyun Shi, David Misch, Stanislav Zak, Megan Cordill, Daniel Kiener

**Affiliations:** 1 Chair of Energy Geosciences, Department of Applied Geosciences and Geophysics, Montanuniversität Leoben, Peter-Tunner-Straße 5, 8700 Leoben, Austria; 2 Erich Schmid Institute of Materials Science, Austrian Academy of Sciences, Jahnstraße 12, 8700 Leoben, Austria; 3 Chair of Materials Physics, Department Materials Science, Montanuniversität Leoben, Jahnstraße 12, 8700 Leoben, Austria

**Keywords:** Nanoindentation, Mudstone, Shale, BIB-SEM, *k*-means, REA, Bootstrapping, Geoenergy

## Abstract

Mudstones and shales serve as natural barrier rocks in various geoenergy applications. Although many studies have investigated their mechanical properties, characterizing these parameters at the microscale remains challenging due to their fine-grained nature and susceptibility to microstructural damage introduced during sample preparation. This study aims to investigate the micromechanical properties of clay matrix composite in mudstones by combining high-speed nanoindentation mapping and machine learning data analysis. The nanoindentation approach effectively captured the heterogeneity in high-resolution mechanical property maps. Utilizing machine learning-based *k*-means clustering, the mechanical characteristics of matrix clay, brittle minerals, as well as measurements on grain boundaries and structural discontinuities (e.g., cracks) were successfully distinguished. The classification results were validated through correlation with broad ion beam-scanning electron microscopy images. The resulting average reduced elastic modulus (*E*_*r*_) and hardness (*H*) values for the clay matrix were determined to be 16.2 ± 6.2 and 0.5 ± 0.5 GPa, respectively, showing consistency across different test settings and indenter tips. Furthermore, the sensitivity of indentation measurements to various factors was investigated, revealing limited sensitivity to indentation depth and tip geometry (when comparing Cube corner and Berkovich tip in a small range of indentation depth variations), but decreased stability at lower loading rates. Box counting and bootstrapping methods were applied to assess the representativeness of parameters determined for the clay matrix. A relatively small dataset (indentation number = 60) is needed to achieve representativeness, while the main challenges is to cover a representative mapping area for clay matrix characterization. Overall, this study demonstrates the feasibility of high-speed nanoindentation mapping combined with data analysis for micromechanical characterization of the clay matrix in mudstones, paving the way for efficient analysis of similar fine-grained sedimentary rocks.

**Supplementary Information:**

The online version contains supplementary material available at 10.1007/s40948-024-00864-9.

## Highlights

- High-speed nanoindentation and k-means reveal micromechanical properties of clay matrix composite in mudstones.
- Clay matrix composite shows consistent modulus and hardness values, with limited sensitivity to indentation depth and tip.
- A small dataset is needed for clay matrix representativeness, but mapping a representative area remains challenging.

**Supplementary Information:**

The online version contains supplementary material available at 10.1007/s40948-024-00864-9.

## Introduction

Mudstones and shales are fine-grained sedimentary rocks that play a crucial role as top seals in various geoenergy applications. Apart from traditional oil and gas exploration, the urgent need for underground storage of energy carriers (e.g., H_2_) and climate-relevant gases (e.g., CO_2_) facilitated extensive research on pore structural and mechanical parameters and their influence on the seal capacity of these rocks (Desbois et al. 2009; Slatt and O’Brien 2011; White et al. 2014; Pan et al. 2021). As a main component in mudstones, the clay matrix forms aggregates of fine-grained and flocculated particles with diameters smaller than 3.9 µm, typically hosting the majority of nanometer- to micrometer-size pores (Nichols 2009; Loucks et al. 2012; Hemes et al. 2013; Houben et al. 2014; Baruch et al. 2015; Zhang et al. 2021). The clay matrix composite—typically mainly clay minerals—can show diverse shapes according to the crystal structure of the respective mineral, as well as depositional processes. In addition to clay minerals, fine particles of amorphous and various shapes surrounding larger brittle grains are also considered part of the clay matrix. The compaction state of the clay matrix is directly linked to its porosity, pore throat distribution, and resulting permeability, which ultimately controls the capillary breakthrough pressure of mudstone barriers and therefore the integrity of the geological reservoir. Moreover, the abundance of ductile clay minerals in mudstones can affect the overall mechanical properties and fracture behaviour of seal rocks (Bourg 2015; Charlet et al. 2017), which in turn controls the risk of seal failure due to microfracturing as a response to various geological processes (e.g., buoyancy pressure from the reservoir, hydraulic fracturing by fluid injection) (Bensing et al. 2023). A key factor to understanding these processes is a geomechanical assessment of the bulk rock. This is routinely done via macroscopic testing of plug or full core samples; however, the geomechanical characterization of mudstones for geoenergy applications is particularly challenging due to the difficulty of preparing well-preserved testing specimen with a defined geometry and the scarcity of preserved core material. Various micromechanical techniques are available for investigating the mechanical properties of the fine-grained, clay mineral-dominated portion in mudstones and shales. These include e.g., atomic force microscopy (AFM) with PeakForce quantitative nano-mechanical mapping (Eliyahu et al. 2015; Graham et al. 2021) and modulus mapping (Zhao et al. 2019). Furthermore, nanoindentation testing is widely applied for local mechanical characterization of materials at small scales (Oliver and Pharr 2004; Hay 2009; Kiener et al. 2009), and is increasingly implemented for use with fine-grained rocks including mudstones, shales and coals as well (Ulm and Abousleiman 2006; Ortega et al. 2007; Bobko and Ulm 2008; Shukla et al. 2013; Bennett et al. 2015; Misch et al. 2018a, b; Lu et al. 2020; Ma et al. 2020; Vranjes-Wessely et al. 2021; Liu et al. 2022). Nanoindentation mapping can provide micromechanical properties of individual phases such as matrix clays. Previous studies showed that drying alteration (e.g., cracking) is likely less intense at micro- compared to macroscale (e.g., Bensing et al. 2023; Skerbisch et al. 2023), making nanoindentation a promising alternative for acquiring representative geomechanical parameters which may later be transferred to classical testing parameters such as unconfined compressive strength (UCS) by empirical relations. However, it is a non-trivial task to determine the micromechanical parameters of clay matrix due to the extreme heterogeneity at the nanometer- to micrometer-scales.

In this study, a workflow was developed using high-speed nanoindentation mapping combined with machine learning data analysis to determine representative mechanical parameters of the clay matrix. The high-speed nanoindentation mapping technique provides hardness (*H*), reduced elastic modulus (*E*_*r*_) and the load–displacement curve for each individual indentation of a mapping array, allowing for the detailed capture of heterogeneity in high-resolution mechanical property maps. Furthermore, broad ion beam-scanning electron microscopy (BIB-SEM) maps were obtained before and after the nanoindentation to correlate the indentation results with direct imaging information. However, classifying the indents as representative for a distinct phase solely based on BIB-SEM images is impossible for extensive mapping arrays, particularly if mixed phases or structural discontinuities are indented, or if no residual imprint can be localized. To address this problem, machine learning-based *k*-means clustering was applied to define representative centroids for the scattered data determined for the soft (clay matrix) and hard (mineral grains) phases (see also Vranjes-Wessely et al. 2021). Grain boundary effects and structural discontinuities can also be excluded with this approach by adding a transitional phase in the *k*-means clustering. The nanoindentation load–displacement curves provide a “mechanical fingerprint” of the tested material and valuable information can be extracted from the shape of the curves (Hainsworth et al. 1996). Therefore, the *k*-means clustering analysis was performed not only on *H* and *E*_*r*_ but also ratio of plastic vs. elastic deformation which was extracted from the shape of each individual on load–displacement curve.

A typical mudstone top seal sample from a major Vienna Basin oil field was selected for testing the proposed workflow. To investigate the reliability of measurements on clay matrix, eight high-speed nanoindentation maps with different experimental settings were obtained. The nanoindentation mapping was performed using both a Berkovich tip and a Cube Corner tip to assess the impact of differently shaped tips on the measurement results and the sensitivity of mechanical parameters to indentation depth and loading rate. As the clay matrix in mudstones is distributed highly variable in 3D within the grain fabric, the covering depth is usually unknown from 2D cross-sections alone. Testing the depth sensitivity can help to test and exclude the “substrate effect”, which refers to the influence of the substrate on the measured mechanical properties of the overlying material (Saha and Nix 2002; Ma et al. 2012; Yang et al. 2020, 2023). In addition, testing penetration at different depths can provide insights into the characteristic length scale of the clay matrix. High testing rates of high-speed nanoindentation mapping enable “high-throughput” testing through fast loading rates; however, some studies have shown that the loading rates can influence the measurement results (Pang et al. 2012; Tarefder and Faisal 2013; Liu et al. 2019; Shi et al. 2019; Wang et al. 2022). Loading rates are also closely related to the strain rate sensitivity, a well-recognized phenomenon in material science (Maier et al. 2011; Hintsala et al. 2018). Therefore, the loading rate sensitivity test was designed to test the effect of fast loading rates. In summary, the indentation sensitivity to (i) tip geometry, (ii) indentation depth, and (iii) loading rate was tested, and the representativeness of the obtained clay matrix properties was discussed.

This study represents an important methodological step towards implementing combined high-speed nanoindentation mapping and machine learning data analysis as a feasible high-throughput tool for the micromechanical characterization of mudstones and similar fine-grained sedimentary rocks.

## Material and methods

### Sample material and preparation

The tested sample was taken from a drill core recovered from a well in the Vienna Basin at a depth of 1628.72 m, representing a facies of relatively homogeneous mudstone top seal above the Middle Miocene (Badenian) sandstone reservoir section “16 TH” (Siedl et al. 2020; Misch et al. 2021). The mineral content of the sample was determined by X-ray diffraction as 31 wt.% quartz, 8 wt.% feldspar, 18 wt.% carbonate, and 39 wt.% clay minerals (Shi et al. 2023). Total organic carbon (TOC) measured using an Eltra Helios C/S analyser is 0.9 wt.%, and the organic matter was found to be thermally immature (Tmax = 417 °C) (Shi et al. 2023). A blocky specimen (ca. 0.7 × 0.5 × 0.5 cm) was cut from the plug which was extracted from the full drill core, and then polished with a Hitachi ArBlade 5000 broad argon ion beam polishing system for a smooth and flat planar surface of ~ 2 mm^2^. The BIB-polished cross-section was Au-coated and imaged with a Tescan Clara field emission SEM before and after the nanoindentation for correlative investigations. Gold is considered a soft metal and the thickness of the gold coating is only a few nanometers (compared to hundreds of nanometers indentation depth), therefore, the influence of the coating on the indentation results can be considered negligible. The SEM images were collected in both backscattered electron (BSE) and secondary electron (SE) modes for compositional (e.g., different mineral grains) and topographic (e.g., residual indentation impressions and pore space morphology) contrast, respectively.

### Nanoindentation mapping

**Fig. 1:**
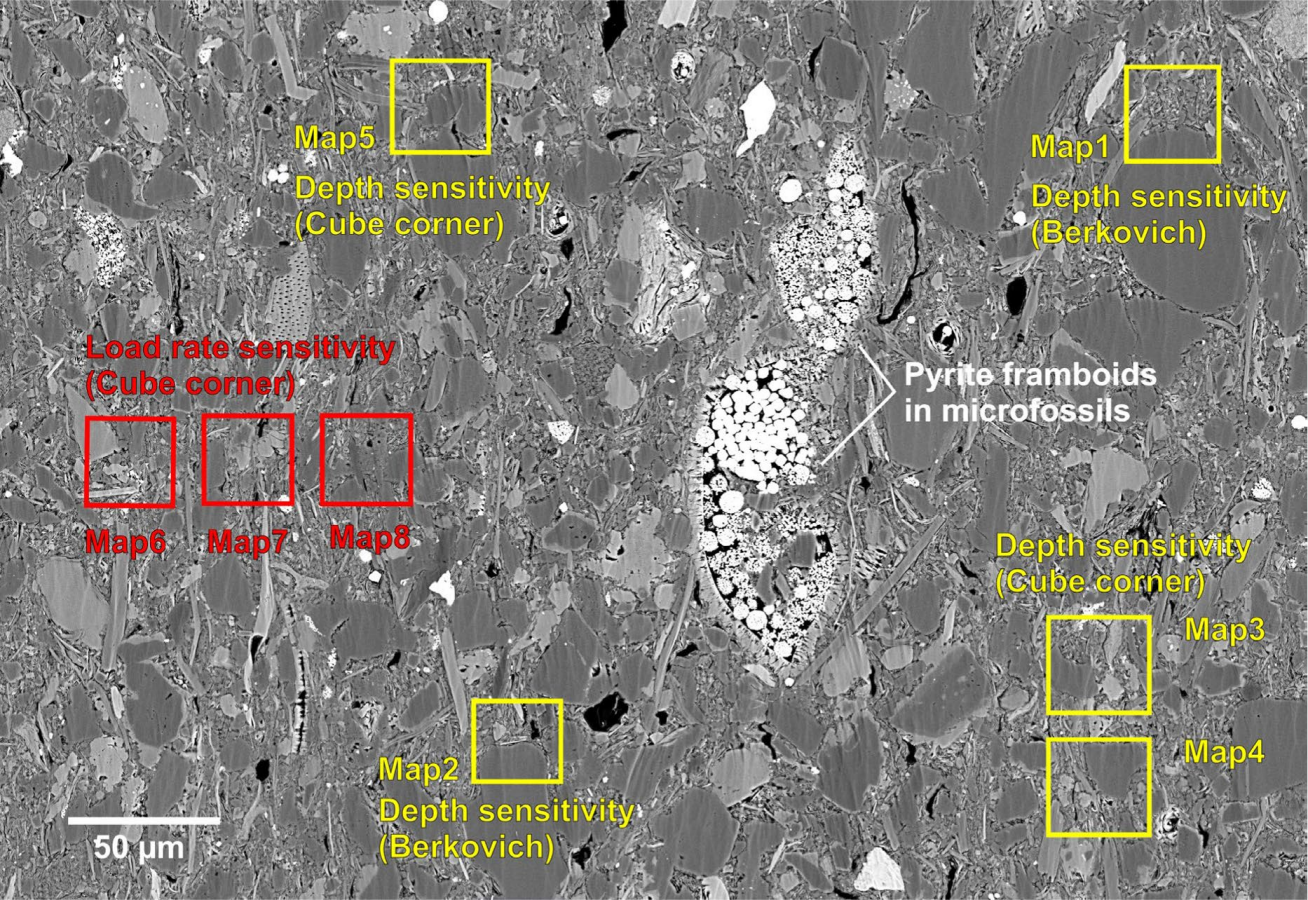
Position of the indentation maps with different experimental setups displayed on SEM (BSE) image. The distinctive structure of the pyrite cement in microfossils helped in locating targeted testing areas during nanoindentation

Nanoindentation property mapping was carried out using a Bruker TS 77 Select Nanoindenter. The measurement setup and data analysis were integrated into the Bruker TS Select Control Software. Before indentation, areas of interest were identified using the light microscope attached to the nanoindenter. It has to be noted that the clay matrix cannot be fully resolved at this magnification; therefore, distinctive features (e.g., large grains, pyrite framboids, microfossils; see Fig. 1) were pre-marked using an external light microscope (Leica DM 4500P) and the pre-indentation SEM images to aid locating the clay matrix area. During the indentation, force and penetration depth were measured continuously. The obtained load–displacement curves were used to determine *E*_*r*_ and *H* according to the Oliver-Pharr method (Oliver and Pharr 1992, 2004).

*H* is the resistance of a material to deformation due to the external force by a sharp indenter, and can be calculated as

1 $$H = \frac{{P_{\max } }}{A}$$

where *P*_max_ is the maximum load applied and *A* is the projected contact area between indenter and material. Instead of imaging the residual indentation impression, *A* is calculated from the tip area function by knowing the geometry of the indenter and the indentation depth at maximal load. A fused silica standard was used for calibrating the tip area function following the Oliver-Pharr method (Oliver and Pharr 1992, 2004).

*E*_*r*_ indicates the deformation occurring in the sample as well as the diamond tip, and is calculated as

2 $$E_{r} = \frac{\sqrt \pi }{2}\frac{S}{\sqrt A }$$

where *S* is the contact stiffness which describes the slope of the unloading curve at peak load.

If the deformation effect of the non-rigid indenter is considered, then the Young’s modulus (*E*) of the sample material can be calculated trough

3 $$\frac{1}{{E_{r} }} = \frac{{1 - v^{2} }}{E} + \frac{{1 - v_{i}^{2} }}{{E_{i} }}$$

where *ν* is the Poisson’s ratio of the sample. While *E*_*i*_ and *ν*_*i*_ are the known Young’s modulus and Poisson’s ratio of the indenter tip, respectively.

A total of eight maps were indented, covering both grain and matrix areas (position is shown in Fig. 1). Each single indentation map covers an array of 7 × 7 indents indented in a serpentine sequence. The indent spacing within each array map was set to 6 μm to fully resolve phase heterogeneities, while still avoiding interference of single indents. Each individual indentation was performed in load-controlled mode with a trapezoidal load function of the same load/hold/unload times. For the indentation testing scheme including the loading–unloading cycles see Fig. 3 in Vranjes et al. (2018) and for the common nanoindentation terminology see Fig. 1 in Hay (2009). To test the effect of different indenter tips and indentation depth, two maps were indented with a Berkovich tip and three maps were indented with a Cube Corner tip, with increasing maximum loads from 500 to 1500 µN to achieve different indentation depths. Both tips are pyramidal tips with a face angle (angle between surface normal of sample and indenter face) of 65.3° for the Berkovich and 35.3° for the Cube Corner (Chudoba et al. 2006). The sharper shape of the Cube Corner allows the tip to penetrate much deeper for the same force. As the loading time was set constantly with 0.3 s, the loading rate calculated by the maximum load divided by the loading time ranges from 1667 to 5000 μN s^−1^ for these five maps (Maps 1–5; Fig. 1). To further test the loading rate sensitivity, three maps (Maps 6–8; Fig. 1) were indented with the Cube Corner tip at a single maximum load of 1000 µN and decreasing loading rates of 6000, 3333, and 1000 μN s^−1^, which was achieved by applying the increasing loading times of 0.16, 0.3, and 1 s, corresponding to a decreasing loading rate. A summary of indentation parameters and settings is given in Table 1.

**Table 1:** List of indentation maps and the respective indentation setting

Array number	Indenter tip	Maximum load (µN)	Load rate (μN s^−1^)
Map1	Berkovich	500–1500	1667–5000
Map2	Berkovich	500–1500	1667–5000
Map3	Cube Corner	500–1500	1667–5000
Map4	Cube Corner	500–1500	1667–5000
Map5	Cube Corner	500–1500	1667–5000
Map6	Cube Corner	1000	6000
Map7	Cube Corner	1000	3333
Map8	Cube Corner	1000	1000

### *k*-means clustering

The *k*-means algorithm in the Python library scikit-learn (Pedregosa et al. 2011) was utilized to classify different phases and determine representative mechanical properties of the clay matrix. The *k*-means approach utilizes an unsupervised machine learning algorithm that clusters *M* samples in *N* dimensions (also referred to as variables/features) into *K* clusters by minimizing the “within-cluster sum of squares” (Hartigan and Wong 1979). The mean value of each cluster is called the cluster “centroid”, calculated repeatedly until a “local” optimum is achieved (Hartigan and Wong 1979). The *k*-means clustering was applied with *H*, *E*_*r*_ and the ratio of plastic to elastic contributions (P/E ratio) as training features to maximize the representativeness of phase-specific data clusters. The proportions of plastic vs. elastic deformation work refer to the area under the loading and unloading parts in the load–displacement curves, respectively (Hainsworth et al. 1996). Prior to clustering, the load–displacement curves were quality-checked to filter out artefacts or invalid results, e.g., where load and displacement do not start at the axis origin or a negative displacement occurs. These erroneous measurements were filtered and marked in red using a self-designed Python script. Grains (e.g., quartz, calcite), grain boundary and other transitional areas, as well as the clay matrix usually have strongly varying mechanical properties ranging from stiff to ductile (see also Vranjes-Wessely et al. 2021). As the clay matrix was in the focus of this study, and all mineral grain constituents show considerably higher *H* and *E*_*r*_ than those of other classes, the number of clusters was set to three (*K* = 3) to capture clay matrix, “others” (including boundary effects and discontinuities), and “grain” (including all brittle mineral grains). Additionally, the Elbow (Thorndike 1953) and Silhouette (Rousseeuw 1987) methods were applied to evaluate the appropriateness of the selected cluster number. The Elbow method is a heuristic approach by plotting the within-cluster sum of squares (also known as inertia) against the number of clusters and identifying the “elbow point” of the curve as the optimal number of clusters. The Silhouette coefficients range from -1 to 1, with a higher silhouette score indicating narrow and well-separated clusters.

To visually validate the *k*-means clustering results, the residual indentation impressions and the indented constituents were identified in the SEM images post-indentation. Additionally, 2D structures (e.g., grain boundaries or pore space) adjacent to the indent locations were documented to interpret the influence of structural heterogeneity on phenomena such as pop-outs and elbows observed in the load–displacement curves.

## Results

### Quality control of the load–displacement curves

**Fig. 2:**
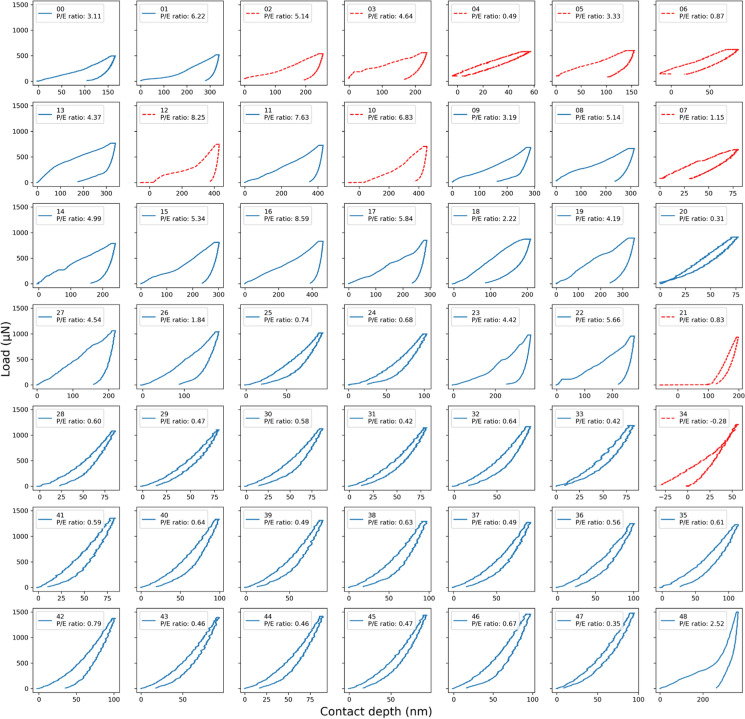
An example of the quality control workflow for load–displacement curves from the depth sensitivity test Map1. The position of individual curves corresponds to the position of the indentation within the indentation map (correlative SEM image see Map1 in Fig. 3). The sequence number of each indentation and the P/E ratio (the ratio of plastic to elastic contributions) are indicated in the subplot legend. Invalid curves are marked in red dash lines after the quality control (e.g., loads and displacements do not start at the zero). P/E ratio—ratio of plastic to elastic contributions

**Fig. 3:**
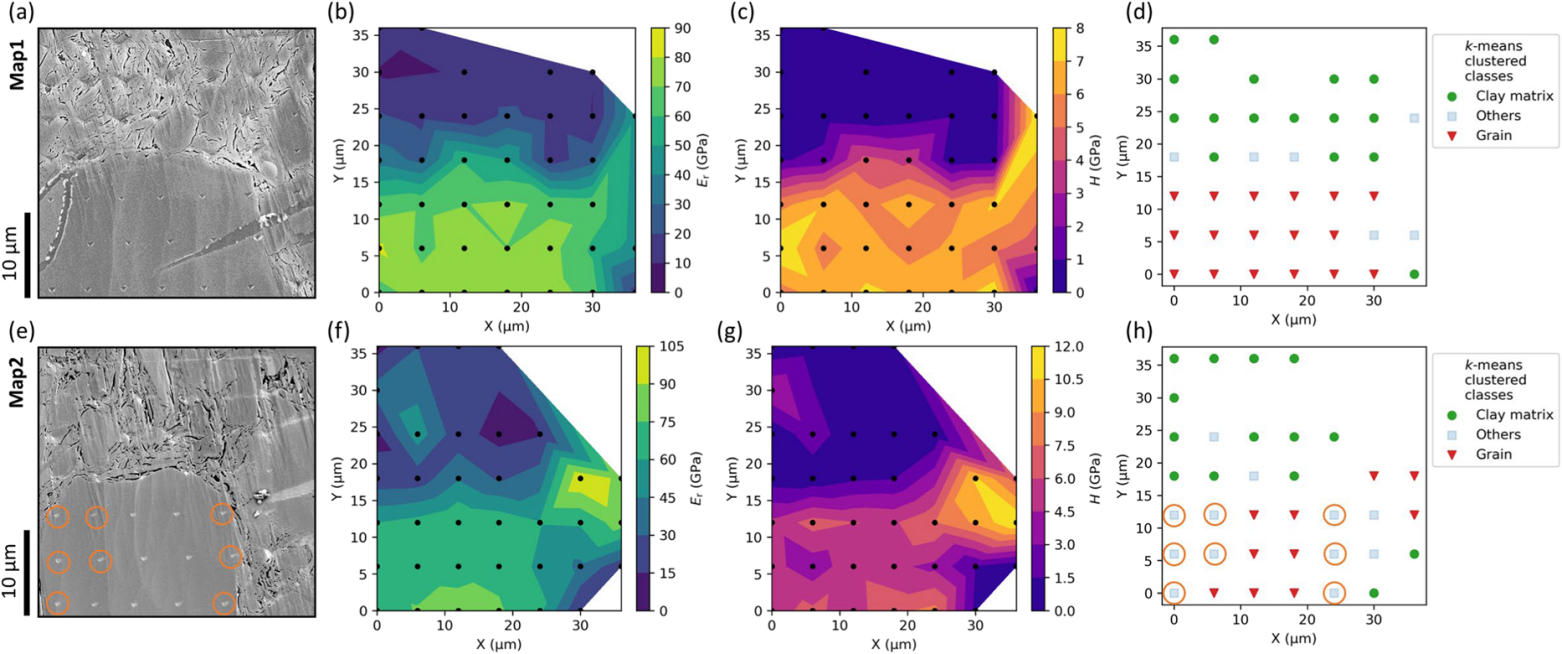
SEM (SE) images, correlative *E*_*r*_ and *H* property maps, and the *k*-means clustering results of Map1 (first row) and Map2 (second row). **a**, **e** SEM images reveal visible indentation imprints after indentation mapping. Scratches on the surface were caused by the removal of the thin gold coating required for imaging. **b**, **f**, **c**, **g** Linear interpolated *E*_*r*_ and *H* property maps. The black dots show the positions of the valid indents. **d**, **h** The *k*-means clustering of the valid indents in colour code. The orange circles in (**e**, **h**) highlight areas of questionable classification of a grain domain as “others”

The load–displacement curves were quality-checked, and the invalid measurements or artefacts were filtered prior to further data analysis, e.g., by *k*-means clustering. Figure 2 shows an example of the quality control workflow for the depth sensitivity test Map1 with all obtained load–displacement curves including the undesirable ones marked in red (quality control of remaining maps is in supplemental). It is noted that the position of individual curves corresponds to the position of the indentation within the indentation map (correlative SEM image see Map1 in Fig. 3). The Python code written for the quality control automatically selected the undesirable load–displacement curves and effectively marked them in red with the filter criteria that loads and displacements do not start at the zero (i.e., displacements offset >  ± 10 nm and load offset >  ± 50 µN) or displacements end in a negative number (offset < − 1 nm). The indentation number 02, 03, 04, 05, 06, 07 were filtered as the loads do not start at zero. The indentation number 10, 12, 21 were filtered due to negative starting displacement records and number 34 was filtered due to negative ending displacement records. These abnormal records could be caused by ambient environment disturbance as well as uncertainty in measurements. The inferior measurements can affect the calculated *H* and *E*_*r*_, and therefore only the reliable indentation measurements were used for the *k*-means clustering. A total of 392 indentation tests were performed on eight maps, of which 318 tests passed the quality control and were considered as valid indentations, yielding a pass rate of 81.1%. The detailed pass rate of each map is described in the following section.

### *k*-means clustering

After filtering out the undesirable curves, the *k*-means clustering was applied to the eight maps indented with different settings described in Table 1.

#### Maps 1–2: depth sensitivity with the Berkovich tip

Map1 and Map2 were indented with the Berkovich tip to test depth sensitivity. The residual imprints in SEM images, *E*_*r*_ and *H* property maps, and the *k*-means clustering results are shown in Fig. 3. SEM images reveal that the residual impressions on the clay matrix are larger than those on the grain area. A total of 39 and 36 indentations passed the quality-check for Map1 and Map2 respectively and were subsequently used for plotting property maps and *k*-mean clustering. The property maps in both Map1 and Map2 show two distinct regions of the indented area: the upper soft/ductile part with low *E*_*r*_ and *H* values and the lower hard/brittle part with high *E*_*r*_ and *H* values, which correspond well with the matrix and grain regions. This can also be associated with the P/E ratios in the curve array in Fig. 2: overall high P/E ratios (e.g., indentation number 01, 11, 16) in the upper part of the curve array indicates a relatively ductile behavior; whereas the low P/E ratios (e.g., indentation number 30, 39, 44) in the lower part of the curve array shows a brittle behavior. The *k*-means clustering of both maps identified the clay matrix correctly. Different classes in Map1 were captured very well, clearly distinguishing the clay matrix and grain area, as well as few indents on the grain boundaries (labelled as “others”). It may be debatable that the *k*-means clustering of Map2 classified the indents at the edge of the lower large grain as “others” (highlighted orange circles in Fig. 3e, h).

**Fig. 4:**
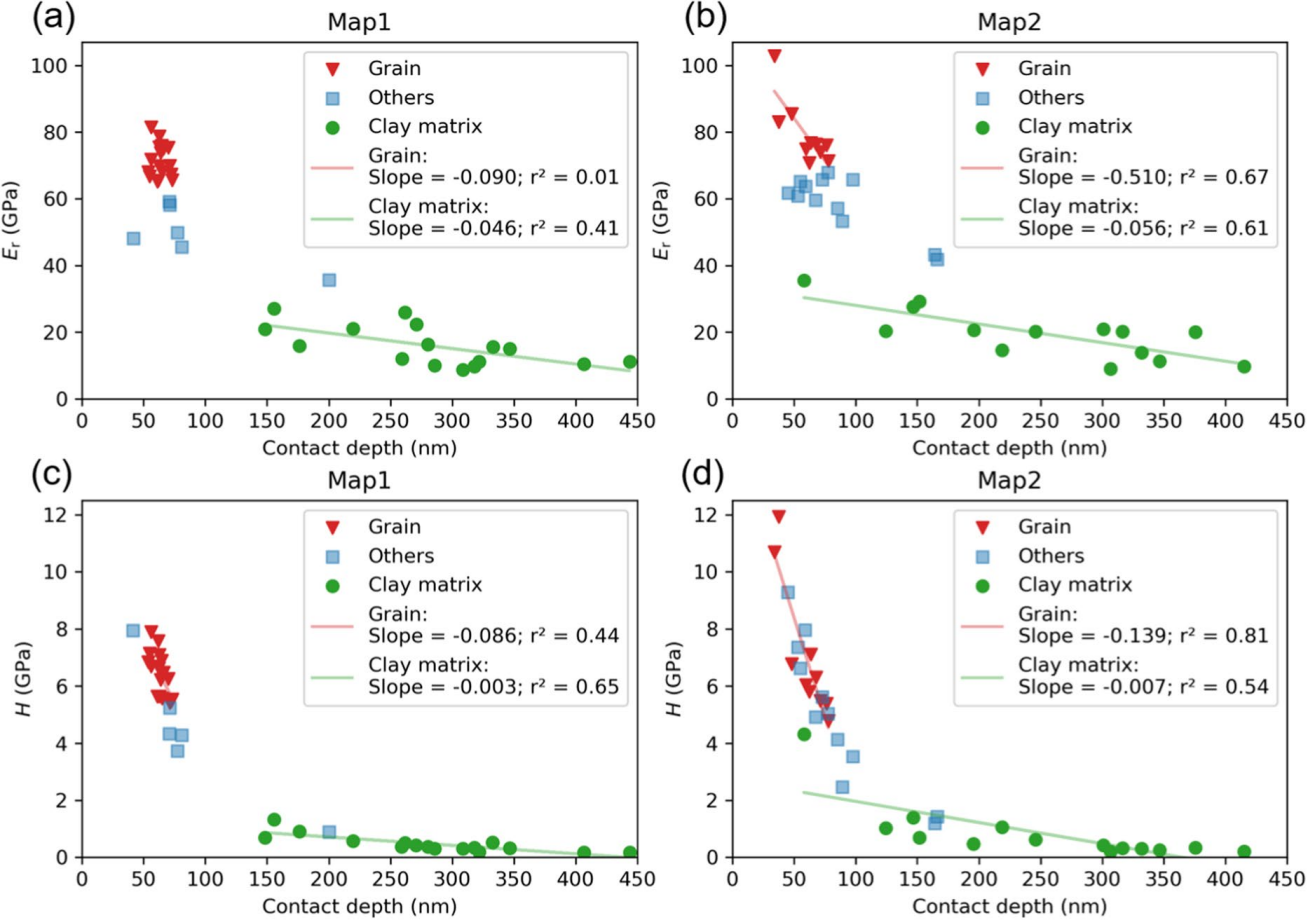
*E*_*r*_- and *H*- contact depth profiles for Map1 (a, c) and Map2 (b, d). The colour code of data points corresponds to the *k*-means clustering results in Fig. 3. r^2^—correlation coefficient

Figure 4 shows the *E*_*r*_ and *H* values plotted against the indentation contact depth for Map1 and Map2. By increasing the load from 500 to 1500 µN, the highest contact depths for two maps were obtained in the “clay matrix” class at about 410–440 nm. The *E*_*r*_ and *H* values of the “grain” and “others” classes decrease sharply over a relatively small depth range. To better describe trends within each class, data points within the same class were linearly fitted, and the corresponding correlation coefficients (r^2^) were calculated. The *E*_*r*_ and *H* values of the “grain” class show a wider scattering with indentation depth for Map1 (r^2^ of 0.01 and 0.44 for *E*_*r*_ and *H*, respectively), whereas these values show a distinct decreasing trend for Map2 (r^2^ of 0.67 and 0.81 for *E*_*r*_ and *H*, respectively). In contrast, the *E*_*r*_ and *H* values of the “clay matrix” class in both maps show a gentle decreasing but remain generally constant from ~ 150 to 400 nm, indicated by moderate correlation coefficients r^2^ of ~ 0.4–0.6 and much lower slopes of − 0.003 to − 0.056 compared to the “grain” class. This suggests that the measured mechanical properties of the clay matrix are less sensitive to a wide range of indentation penetration depths than those of the other phases.

#### Maps 3–5: depth sensitivity with the Cube Corner tip

**Fig. 5:**
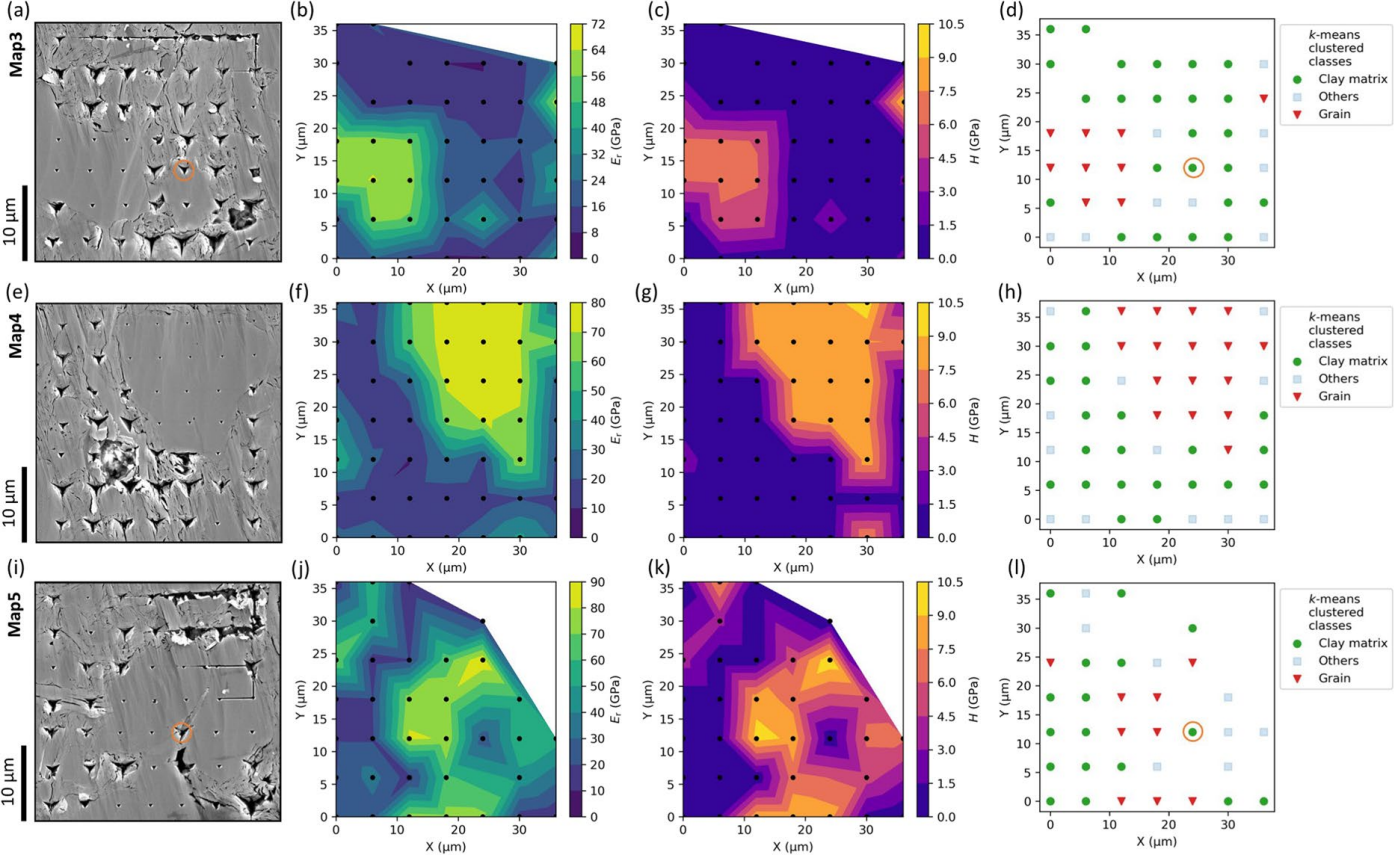
SEM (SE) images, correlative *E*_*r*_ and *H* property maps, and the *k*-means clustering results of Map3 (first row), Map4 (second row) and Map5 (third row). **a**, **e**, **i** SEM images reveal visible indentation imprints after indentation mapping. Linear features visible on the slightly uneven sample surface are scratches from the moving nanoindentation tip. A grain was broken out during the indentation, leaving a circular damage on the surface in (**e**). **b**, **f**, **j**, **c**, **g**, **k** Linear interpolated *E*_*r*_ and *H* property maps. The black dots show the positions of the valid indents. **d**, **h**, **l** The *k*-means clustering of valid indents in colour code. The orange circles in (**a**, **d**, **i**, **l**) highlight possibly debatable classification

Map3, Map4 and Map5 were indented with the Cube Corner tip to test depth sensitivity. Figure 5 shows the residual imprints in SEM images, *E*_*r*_ and *H* property maps, and the *k*-means clustering results. The residual impressions on the clay matrix and the interparticle area are larger than those on the grain area, and are visually much deeper compared to those left by the Berkovich tip (Figs. 3a, e, 5a, e, i). After the quality-check of the indentation curves, 42, 49 and 34 indentations were selected for plotting property maps and *k*-means clustering of Map3, Map4 and Map5, respectively. The *E*_*r*_ and *H* property maps provide a very good local mechanical characterisation of the clay matrix and grain domains, with the shape of the grain being clearly highlighted (Fig. 5b, c, f, g, j, k). In general, the *k*-means clustering successfully distinguishes clay matrix and larger grains, with few indents on smaller grains and grain boundaries identified as “others”. However, some indents labelled as “clay matrix” may be debatable (highlighted orange circles in Fig. 5a, d, i, l), as these indents are in proximity to grain boundaries.

**Fig. 6:**
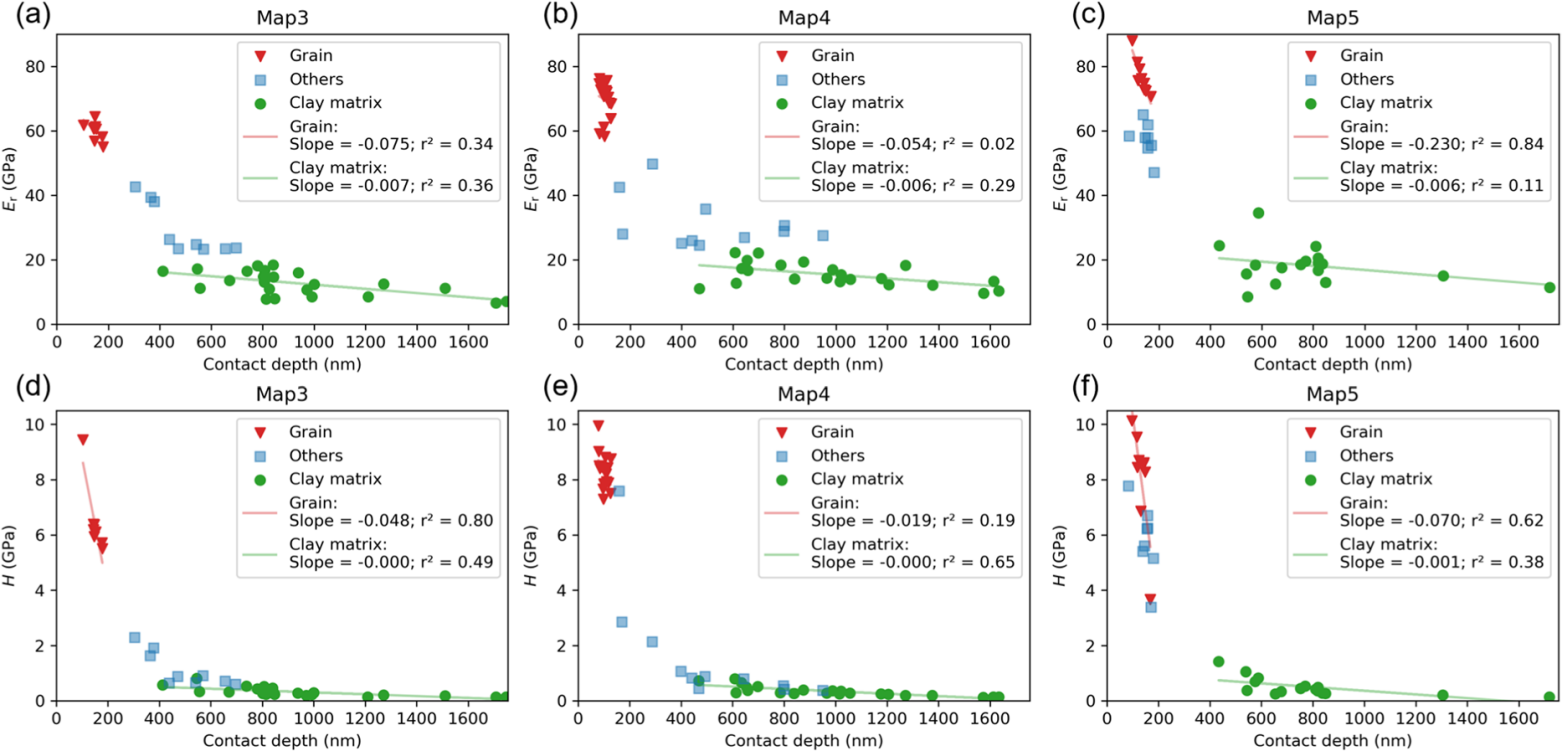
*E*_*r*_- and *H*- contact depth profiles for Map3 (**a**, **d**), Map4 (**b**, **e**) and Map5 (**c**, **f**). The colour code of data points corresponds to the *k*-means clustering results in Fig. 5. r^2^—correlation coefficient

Figure 6 displays the *E*_*r*_ and *H* values plotted against the indentation contact depth for Map3, Map4, and Map5. The *E*_*r*_- and *H*- contact depth profiles from the Cube Corner and Berkovich tips are identical for the three clustered classes: (i) the high *E*_*r*_ and *H* values at shallow depths are from the “grain” class, (ii) the low *E*_*r*_ and *H* values at the higher depths are from the “clay matrix” class, and (iii) the “others” class lies in-between and has moderate *E*_*r*_ and *H* values. The deepest Cube Corner contact depths of all three maps were obtained in the clay matrix at about 1630–1750 nm, which is much deeper compared to the Berkovich indentations and correlates well with the visually more pronounced impressions in the SEM images. The *E*_*r*_ and *H* values of the “grain” and “others” classes decrease with increasing depth in a relatively small range, indicated by high slopes of − 0.019 to − 0.230. As the indentation depth increases from ~ 500 to 1750 nm, the *E*_*r*_ and *H* values of the “clay matrix” class also show some fluctuations but remain generally constant (low slopes of − 0.000 to − 0.007), in a similar pattern to those indented with the Berkovich tip. Overall, this suggests that the clay matrix shows less depth-sensitivity during nanoindentation with both tips.

**Fig. 7:**
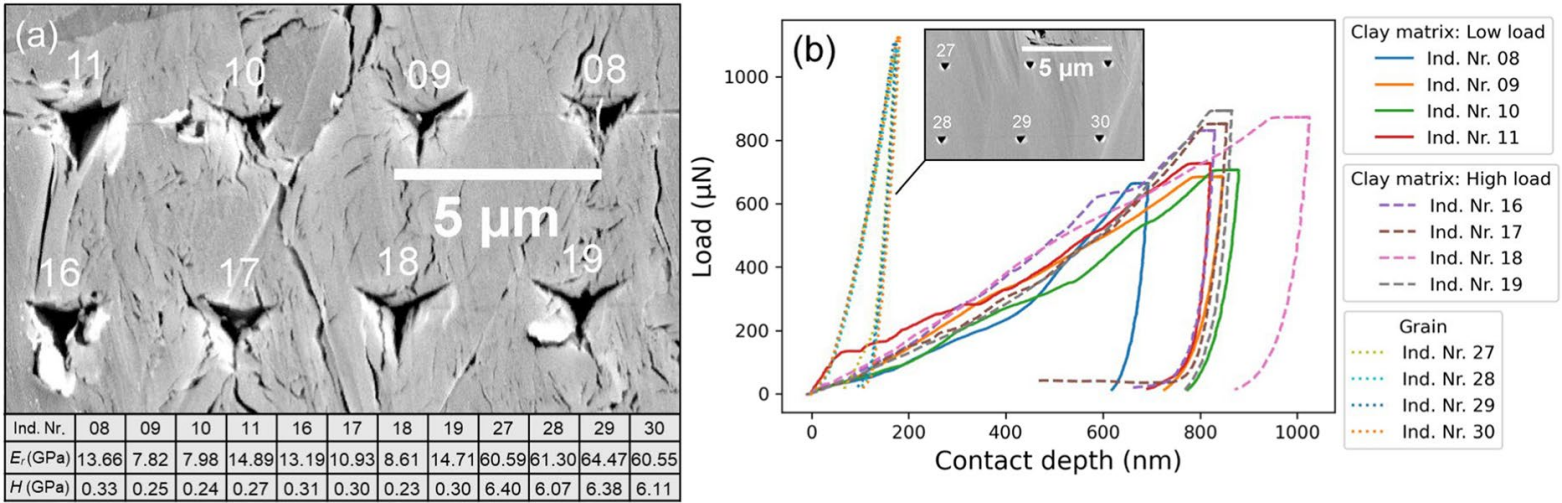
Remnant of nanoindentation imprints and corresponding load–displacement curves from Map3. **a** Close look of the indents on clay matrix (also see Fig. 5a). **b** Load–displacement curves of the indentations in (**a**). Inset shows the imprints on the rigid grain area corresponding to the steep curves. Attached table lists the obtained *E*_*r*_ and *H* for indents in (**a**) and insert in (**b**). Ind. Nr.—indent number

Figure 7 shows typical indentation imprints on different phases observed on the SEM images for Map3, correlating with their respective load–displacement curves. As a serpentine sequence of indentations with increasing maximum load was performed, two categories were found for the clay matrix: an upper row (indent number 08, 09, 10, 11) with lower loads and a lower row (indent number 16, 17, 18, 19) with higher loads (Fig. 7a). Despite a gradual increase in the maximum loads, most of the load–displacement curves exhibit an identical shape, which is significantly different from the indentations observed on the rigid grain area (insert in Fig. 7b). The load–displacement curves for grain show highly overlapping, smooth, and steep loading sections, whereas those for the clay matrix displays slightly wavy and fluctuating loading sections, indicative of its porous internal structure. Except for the indent numbers 08 and 18, the load–displacement curves show slightly deviations from their respective load categories, resulted in slightly shifted mechanical values.

#### Maps 6–8: load rate sensitivity with the Cube Corner tip

**Fig. 8:**
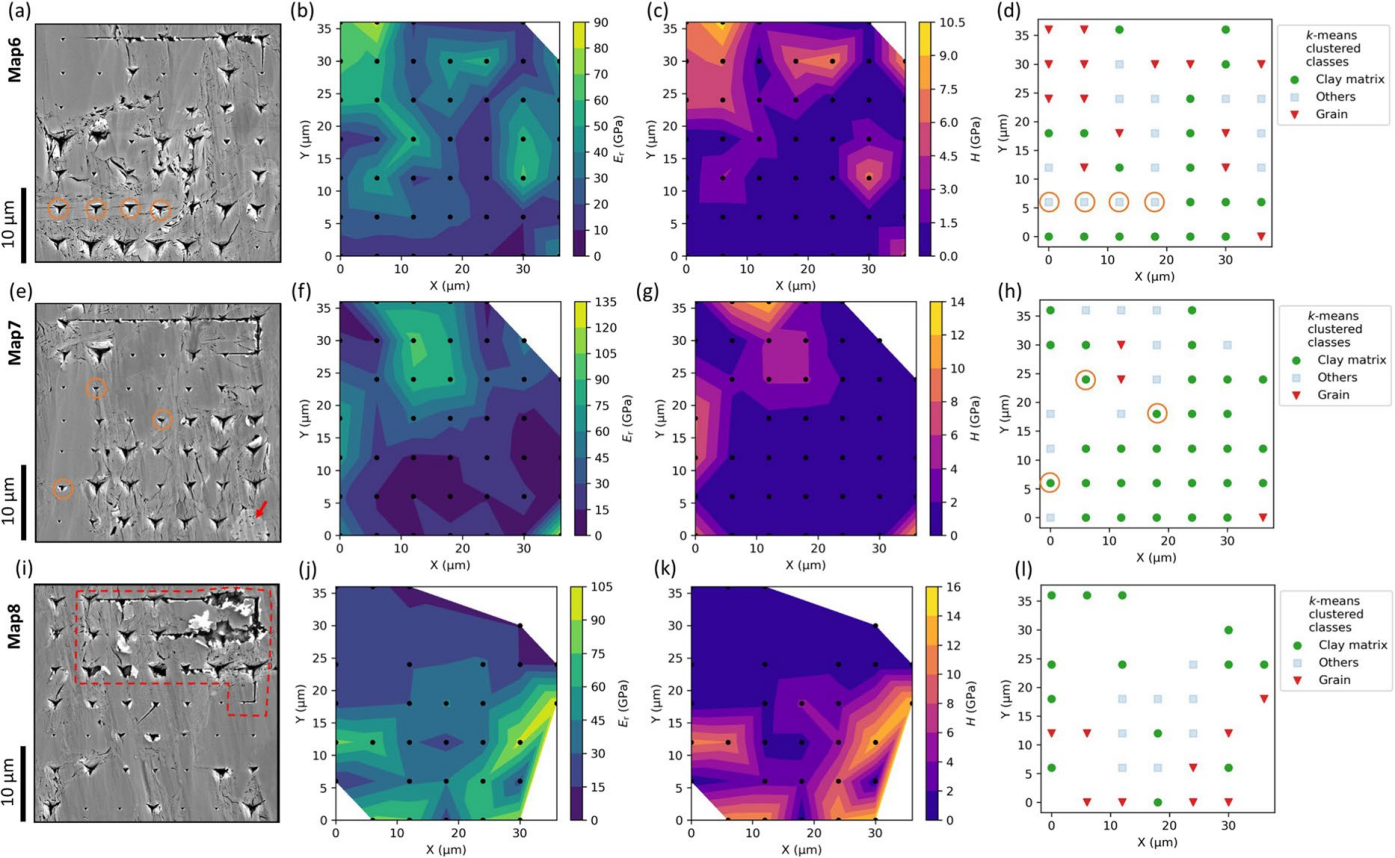
SEM (SE) images, correlative *E*_*r*_ and *H* property maps, and the *k*-means clustering results of Map6 (first row), Map7 (second row) and Map8 (third row). **a**, **e**, **i** SEM images reveal visible indentation imprints after indentation mapping. Linear scratch damage on the surface is the move path of the nanoindentation tip. The red arrow in (e) indicates the indent on a pyrite framboid. The red dashed line in (**i**) encloses an area of strong surface damage. **b**, **f**, **j**, **c**, **g**, **k** Linear interpolated *E*_*r*_ and *H* property maps. The black dots show the positions of the valid indents. **d**, **h**, **l** The *k*-means clustering of the valid indents in colour code. The orange circles in (**a**, **d**, **e**, **h**) highlight possibly debatable classification

Figure 8 shows the results of Map6, Map7, and Map8 for the loading rate sensitivity test with the Cube Corner tip. In Map6, 46 indents were selected after the quality control. *E*_*r*_ and *H* property maps display several brittle/hard regions which correspond to the grain areas visible in the SEM images (Fig. 8a–c). The *k*-means clustering shows an overall good classification of the clay matrix and grain areas. Interestingly, four indents on the long clay mineral particle in the lower left of the map are classified as “others” (highlighted orange circles in Fig. 8a, d). This indicates that the intact clay mineral particle is mechanically stronger than the agglomerates of randomly oriented smaller clay particles. In Map7, correlative SEM images reveal two grain areas on the left and upper middle of the map, corresponding well with the *E*_*r*_ and *H* property maps (Fig. 8e, f, g). Map7 with 43 valid indents shows that most of the indents are classified as “clay matrix”, although the classification of few indents may be debatable (Fig. 8e, h). The last indent on the lower right of the map indented into a pyrite framboid (Fig. 8e), yielding the highest *E*_*r*_ of 121.58 GPa and a high *H* value of 10.94 GPa. The indentation impression on the pyrite framboid is barely visible as its indentation contact depth is 100 nm compared to ~ 500–1000 nm for the clay matrix. Map8 with the lowest loading rate of 1000 μN s^−1^ shows more surface damage along the indentation path in the upper two rows and more pronounced pile-up material piled around the indention imprints (red dashed line in Fig. 8i). After the quality control of the load–displacement curves, only 29 indents were found valid for further data processing. *E*_*r*_ and *H* property maps poorly represent the local mechanical characteristics due to many missing indents (Fig. 8j, k). The *k*-means clustering result of Map8 are also statistically less representative.

**Fig. 9:**
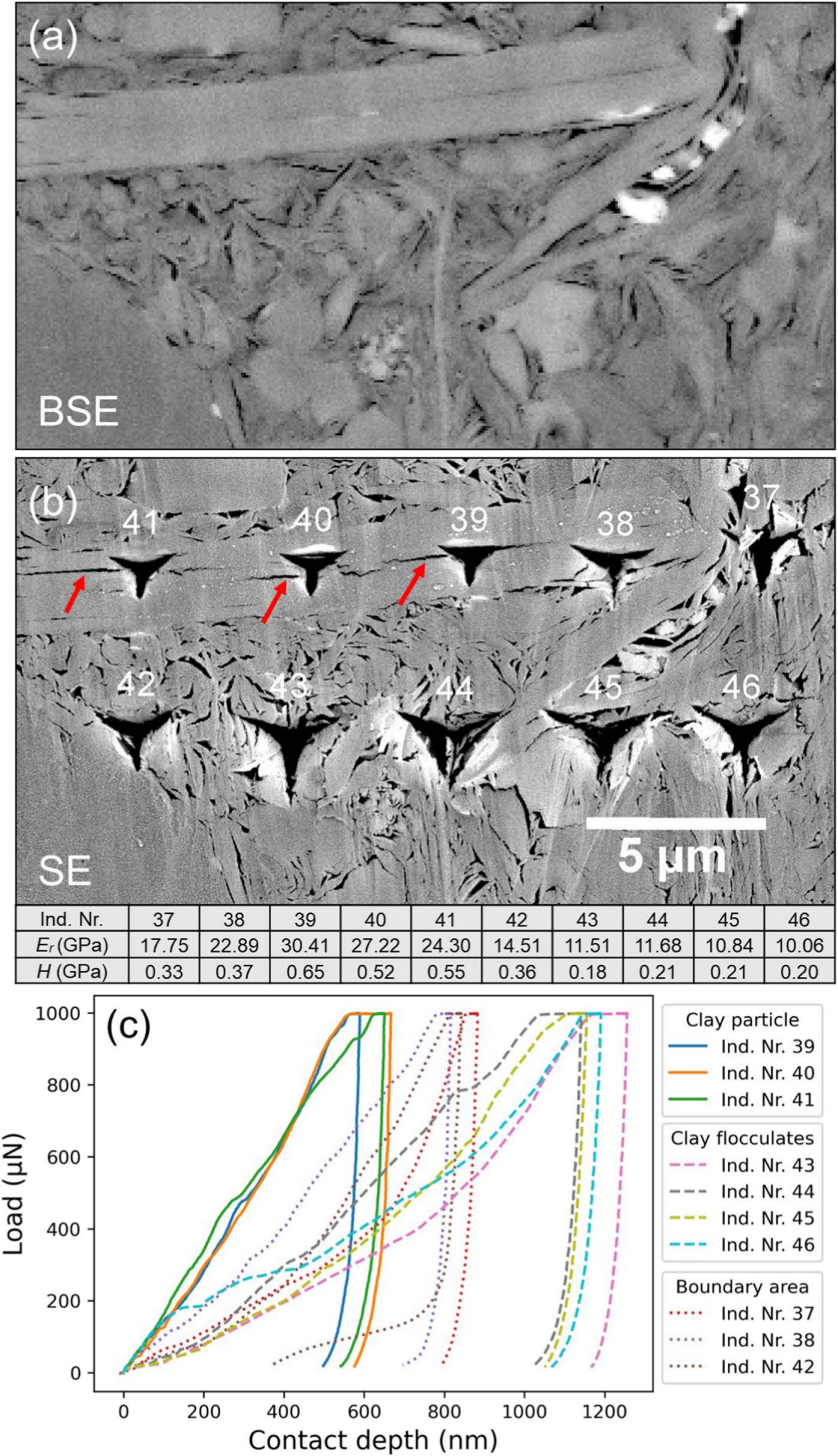
Image correlation and corresponding load–displacement curves from Map6. **a**, **b** SEM (BSE & SE) images of the clay matrix before and after nanoindentation (also see Fig. 8a). **c** Load–displacement curves of the indentations in (**b**). The red arrows indicate indentation-induced opening along crystal sheets. Attached table lists the obtained *E*_*r*_ and *H*. BSE- backscattered electron. SE—secondary electron. Ind. Nr.—indent number

Figure 9 shows a careful analysis of the indents on the clay mineral particle and clay aggregates in Map6 with SEM images and their respective load–displacement curves. Their distinct mechanical behaviour suggests that clay minerals can be categorized into two primary structures: clay aggregates and large clay particles/platelets. During nanoindentation, the clay matrix composed of aggregates experienced a higher degree of ductile deformation compared to the individual, larger clay mineral platelets, leading to larger imprints and deeper indentation depths. Indentation on the clay mineral platelets caused the separation of crystal sheets along cleavage planes (highlighted with red arrows in Fig. 9b). The load–displacement curves for the indents along the boundary area between the clay particles and surrounding aggregates (indent numbers 37, 38), as well as between the aggregates and a brittle grain (indent number 42), plot in-between the curves of individual clay mineral particles and aggregates. The greater mechanical strength determined for the individual clay mineral platelet also supports the accuracy of the *k*-means clustering, which classified the clay particle as “others” (highlighted orange circles in Fig. 8a, d).

### Statistical distribution of phase-specific micromechanical properties

Table 2 summarizes the *k*-means clustered results including mean and standard deviation (SD) values of *E*_*r*_, *H* and P/E ratio for different phases in eight indentation maps. The *k*-means clustering shows distinct mechanical characteristics of the “clay matrix”, “others” and “grain” classes with the increasing *E*_*r*_ values of 16.2 ± 6.2, 42.9 ± 16.1, 71.2 ± 12.7 GPa, respectively, and the increasing *H* values of 0.5 ± 0.5, 3.2 ± 2.8, 7.4 ± 2.4 GPa, respectively. Map1 and Map2 with the Berkovich tip yielded much lower mean P/E ratio for all three classified phases compared to the maps with the Cube Corner tip. The overall high SD values suggest that the classified classes may contain multiple components. Nevertheless, the classification was successful in assigning clusters with distinct mechanical behaviour, as confirmed by SEM images. For the “clay matrix” in all eight maps, the mean *E*_*r*_ values range from 13.12 to 19.53 GPa and the* H* values vary from 0.32 to 0.83 GPa. Data points in the “others” class show large variability with mean *E*_*r*_ and *H* values ranging from 28.35 to 64.10 GPa and from 0.83 to 6.22 GPa, respectively. The “grain” class in all maps has the highest mean *E*_*r*_ and *H* values ranging from 56.35 to 96.48 GPa and from 5.73 to 11.82 GPa, respectively, yielding the lowest relative standard deviation. Map7 has the highest mean value of *E*_*r*_ and the highest SD values of *E*_*r*_ and* H* in the “grain” class, which is due to the indent on the stiff framboidal pyrite domain (Fig. 8e).

**Table 2:** Summary of the *k*-means clustering results for different phases on each map. Mean *E*_*r*_ and *H* values, corresponding standard deviation (SD) values and the number of valid indents (n) on different phases are listed. P/E ratio—ratio of plastic to elastic contributions.

Phases	Nanoindentation map	*E*_*r*_ (GPa)		*H* (GPa)		P/E ratio		n
		Mean	SD	Mean	SD	Mean	SD	
Clay matrix	Map1	15.9	6.0	0.5	0.3	4.7	1.9	16
	Map2	19.5	7.6	0.8	1.1	3.9	2.0	14
	Map3	12.7	3.7	0.3	0.2	11.6	3.7	24
	Map4	15.1	3.4	0.3	0.2	10.9	4.3	21
	Map5	18.2	6.0	0.5	0.3	10.8	4.7	17
	Map6	13.1	3.2	0.4	0.5	12.0	5.6	18
	Map7	18.5	8.2	0.7	0.5	9.2	4.6	30
	Map8	18.4	5.6	0.6	0.3	10.8	4.9	11
	All maps	16.2	6.2	0.5	0.5	9.5	4.9	151
Others	Map1	49.4	8.7	4.4	2.3	1.2	1.6	6
	Map2	58.8	8.7	5.0	2.6	1.2	0.9	12
	Map3	29.5	8.1	1.1	0.6	6.6	1.3	9
	Map4	30.7	8.1	1.6	2.0	7.2	4.4	12
	Map5	57.3	5.3	5.8	1.3	2.0	1.0	8
	Map6	28.4	6.0	0.8	0.6	10.2	4.3	14
	Map7	64.1	9.5	6.2	3.7	3.2	2.2	9
	Map8	35.5	7.6	2.4	1.4	5.0	3.3	9
	All maps	42.9	16.1	3.2	2.8	5.1	4.3	79
Grain	Map1	71.4	5.0	6.4	0.8	0.5	0.1	17
	Map2	79.1	9.5	7.0	2.4	0.7	0.2	10
	Map3	60.0	2.8	6.4	1.2	1.6	0.3	9
	Map4	69.6	5.9	8.3	0.7	1.3	0.3	16
	Map5	76.8	5.3	8.1	1.9	1.8	1.0	9
	Map6	56.4	10.1	5.7	2.1	2.5	1.5	14
	Map7	96.5	17.6	6.0	3.3	3.9	1.7	4
	Map8	82.0	11.3	11.8	1.8	1.3	0.4	9
	All maps	71.2	12.7	7.4	2.4	1.5	1.1	88

## Discussion

### Performance of the *k*-means clustering

The *k*-means clustering was performed for each indentation maps and proven to be effective and efficient in classifying the mechanical response into three distinct classes: “clay matrix”, “grain”, and “others” (Table 2). The clustering results correlated well with the SEM images (Figs. 3, 5, 8), despite different nanoindentation test settings and indenter tips (Table 1). This successful clustering can be attributed to inherent differences in the characteristics of the designated classes.

**Fig. 10:**
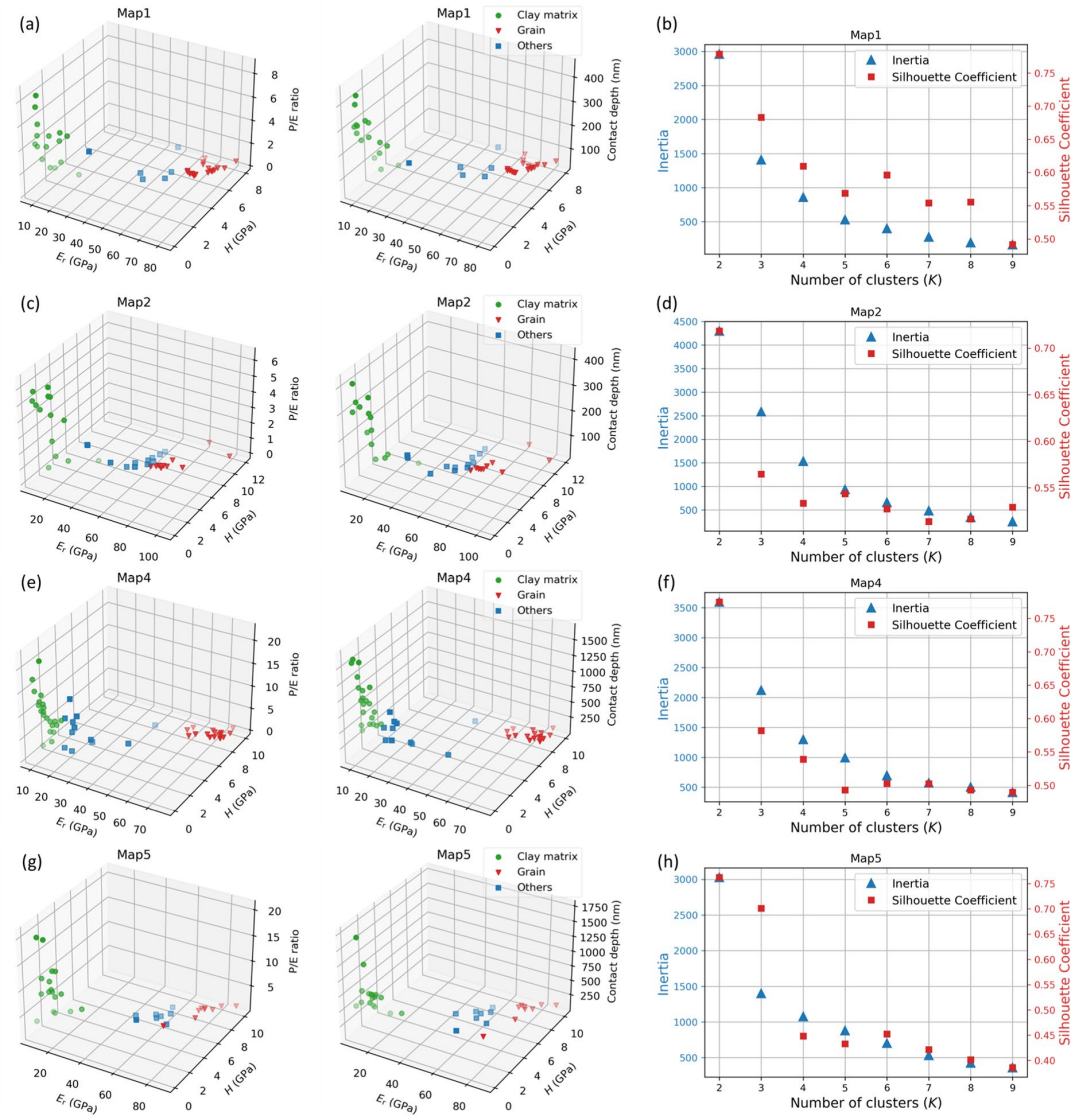
*k*-means clustering results in 3D feature space and metrics plots for evaluating the number of clusters for Map1, Map2, Map4 and Map5. **a**, **c**, **e**, **g** *E*_*r*_ and *H* plot against P/E ratio and contact depth in 3D to reveal the cluster distribution in feature space. **b**, **d**, **f**, **h** Dual axis plots of Inertia and Silhouette coefficient vs. number of clusters

The feature space plots for four exemplary maps show that within each cluster, the data points display high similarity in the collective attributes of *H*, *E*_*r*_, P/E ratio, and contact depth (Fig. 10). The “clay matrix” class typically has low *H* and *E*_*r*_ values, and high P/E ratio and contact depth, while the “grain” class has high *H* and *E*_*r*_ values, and low P/E ratio and contact depth. The “others” fell between these two categories, exhibiting a more random parameter distribution. Ideally, greater separation between clusters in the feature space leads to more unambiguous *k*-means clustering. The significant distance between the “clay matrix” and “grain” classes facilitated a clear clustering tendency, contributing to the overall effectiveness of the *k*-means clustering approach.

However, some challenges were encountered with the “others” class. Some data points within this class display spatial separation (Fig. 10a) or overlap with the “grain” (Fig. 10c) or “clay matrix” (Fig. 10e) classes in the feature space. This dispersed nature of the "others" class posed a challenge to the clustering task, which is also reflected in the metrics for evaluating the number of clusters (Fig. 10b, d, f, h). The evaluation metrics such as “Inertia” and “Silhouette Coefficient” were used to determine the optimal number of clusters. Usually, the optimum cluster number is indicated by an “elbow” point in the Inertia curve where the metric points visibly bend to flat, as well as a relatively high Silhouette Coefficient. For most maps, there is no clear “elbow” point in the Inertia curve and the Silhouette Coefficient drops rapidly after *K* = 2 (Fig. 10b, d, f), suggesting no optimum cluster number. Only Map5 shows that *K* = 2 might be a better cluster number than *K* = 3, as the Inertia drops from a high slope to a low slope, and the Silhouette Coefficient still remains relatively high (Fig. 10h). Overall, the metrics plots indicate that determining an optimal number of clusters for such heterogeneous material with complex mineral phases is challenging.

Considering *K* = 2 may be ideal when the objective is to simply differentiate between hard (brittle grains) and soft (clay matrix) regions. However, only *K* = 3 fulfills the task of separating the soft clay matrix from the brittle mineral grains and while sufficiently excluding grain boundaries, cracks, etc. to achieve representative phase-specific properties (Vranjes-Wessely et al. 2021). In this case, the “others” class serves as a smooth transition or buffer between the “clay matrix” and “grain” classes. It should be noted that when using the predefined *K* = 3, some data points near the feature boundary could exhibit equidistant or close proximity to multiple centroids. Consequently, to achieve a balanced distribution across the three classes, such points were forced to be assigned to the nearest cluster, leading to potential ambiguous assignment and improper classification in certain cases (Figs. 3e, 9, 10c). Nevertheless, in most cases, the inclusion of the “others” class provides an additional data interval and improve the accuracy of clustering the clay matrix.

Previous nanoindentation studies on shales and mudstones have identified distinct mechanical phases (Zeszotarski et al. 2004), and the utilization of *k*-means clustering has proven to be an efficient approach for geological reasoning of micromechanical property distributions (Shukla et al. 2013; Vranjes-Wessely et al. 2021; Liu et al. 2022; Puchi-Cabrera et al. 2023). This study aligns with those previous findings and supports the use of *k*-means clustering for the micromechanical characterization of the clay matrix in mudstones. The clustering results obtained in this study were consistent with the findings from image correlation, further validating the reliability of the approach. Moreover, the obtained *E*_*r*_ values of 16.2 ± 6.2 GPa and *H* values of 0.5 ± 0.5 GPa for the clay matrix based on the *k*-means clustering lie within similar ranges as reported in previous nanoindentation studies. For example, Bobko and Ulm (2008) reported moduli ranging from 16 to 25 GPa and hardness values from 0.57 to 0.62 GPa for the porous clay phase of organic-free shale samples, with variations depending on indentation direction (parallel or perpendicular to the bedding plane) and indentation depths. However, Abedi et al. (2016) state that organic-rich shales exhibit higher moduli ranging from 48.5 to 63.1 GPa and higher hardness values ranging from 2.2 to 2.6 GPa for the clay phase, depending on indentation directions but showing no systematic influence of thermal maturity and total organic carbon content. Compared to the immature and organic-lean samples used in this study, Yang et al. (2020) also reported higher Young's moduli and hardness values of 30 and 1.5 GPa, respectively, for clay minerals in organic-rich overmature shales. These results emphasize the importance of formation-specific investigations to capture the inherent geologic variability.

**Fig. 11:**
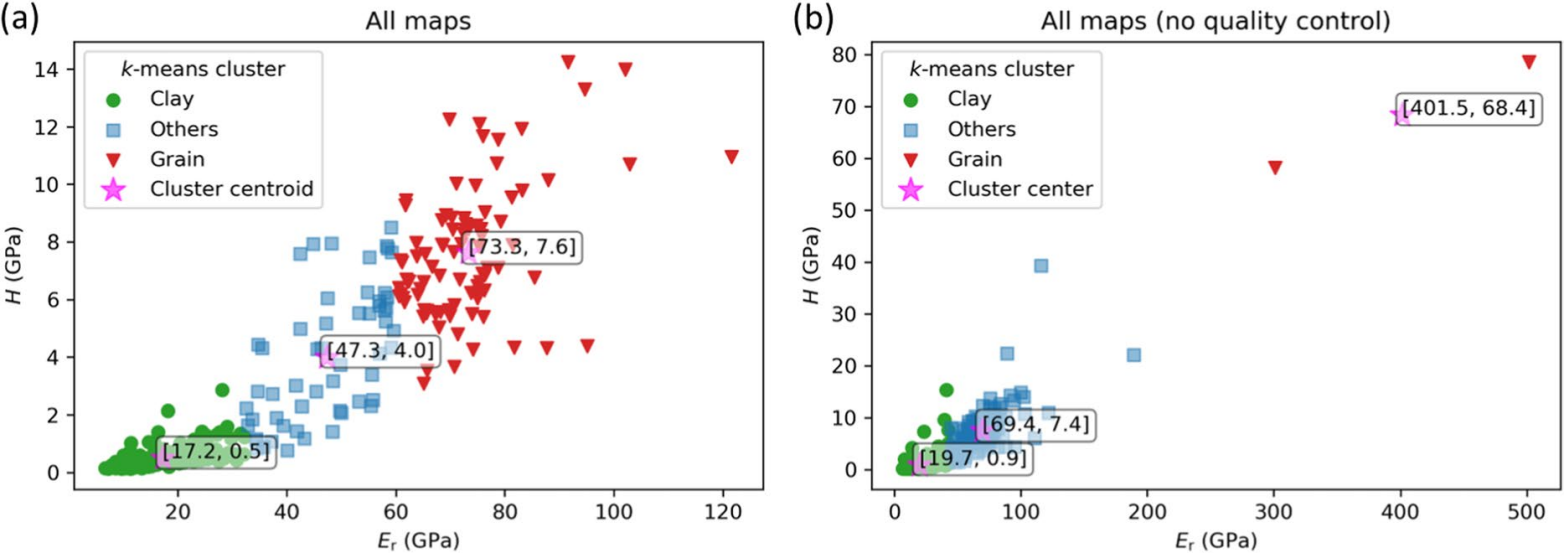
*k*-means clustering including data from all eight maps. **a** *k*-means clustering using only valid indents. **b** *k*-means clustering using all indents without preceding quality control based on load–displacement curves

The *k*-means clustering approach was additionally applied to all valid indents in the eight distinct maps (Fig. 11a), yielding comparable results to the *k*-means clustering applied separately to each individual map. The representative centers for the clustering of the cumulative data set are close to those calculated based on the individual maps (Fig. 11a, Table 2). The overall good match shows that the *k*-means clustering is consistent and well-balanced in both scenarios (overall vs. individual). It is noted that the overall *k*-means clustering used only *E*_*r*_ and* H* as training features, compared to the clustering of individual maps which also included the P/E ratio. The matching results of the two different clustering approaches suggests that *E*_*r*_ and* H* sufficiently represent the inherent mechanical properties of the tested phases, while the P/E ratio is more influenced by the application of different indentation tips. This difference in P/E ratio can be assigned to the representative strain of different tips which is dependent on the half apex angle of the tip: ~ 7% strain for the Berkovich tip and ~ 22% strain for the Cube Corner tip (Leitner et al. 2017; Tabor 2000). The *k*-means clustering was furthermore applied to the overall data set including all data points without any quality control of the load–displacement curves (Fig. 11b), yielding a significant shift of the cluster centers of the “others” and “grain” classes. Interestingly, the cluster center of the “clay matrix” class did not change considerably compared to the quality-checked clustering results, presumably due to the clearly distinct (lower) *E*_*r*_ and* H* of the clay mineral matrix compared to all other grain components. Nevertheless, the comparison of pre- and post-correction clustering still highlights the importance of the quality control of the load–displacement curves prior to the *k*-means clustering.

### Indentation sensitivity

**Fig. 12:**
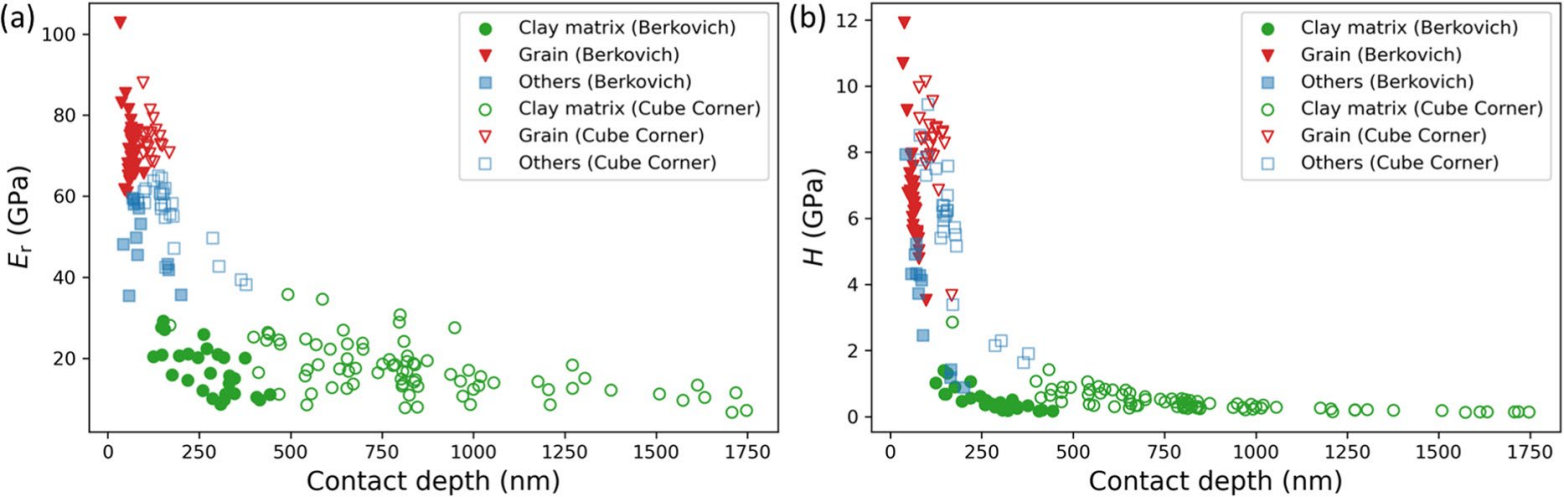
**a** *E*_*r*_- and **b** *H*- contact depth profiles for Maps 1–5 with the Berkovich and Cube Corner tips. The colour code of data points corresponds to the *k*-means clustering results in Figs. 3, 5

The *E*_*r*_ and *H* values of the “clay matrix” class obtained by both Berkovich tip and Cube Corner tip remained relatively consistency over a large contact depth range (Figs. 4, 6), indicating a limited sensitivity of the clay matrix to the contact depth. Despite the significant difference in contact depths achieved by the two tips (maximum 450 vs. 1750 nm) (Fig. 12), the measured mechanical properties were comparable and reliable, indicating properly calibrated tip area functions. The difference in contact depths can be attributed to the geometry of the tips, with the Cube Corner tip being sharper and penetrating to greater depths with the same applied force. Vice versa, a Berkovich tip samples a much larger volume at the same indentation depth compared to a Cube Corner (Kiener et al. 2009). Yang et al. (2020) reported a depth larger than 300 nm as being sufficient for the determination of inherent mechanical properties in clay composites using the dynamic nanoindentation technique with a Berkovich tip. In this study, the relatively consistent mechanical response of the clay matrix to the indentation contact depths ranging from ~ 300 to 1750 nm with both types of tips aligns with these previous findings. This depth interval suggests a characteristic length scale of the clay matrix that is free from the “substrate effect”. Despite clay matrix being composed of aggregates, clay particles, and mineral fragments, it is still possible to obtain stable mechanical properties for this complex material.

In contrast, the classes “others” and “grain” vary considerably over a relatively narrow short depth range and are hardly uniform, possibly due to indentation into different mineral types (e.g., quartz, calcite, and pyrite), grain sizes and shapes, as well as varying proximity to grain boundaries. Indentation on single grains (e.g., Map1, Map2 and Map4) shows a decrease in *H* as the indentation depth increases, indicating a possible indentation size effect, which is well acknowledged in material science (Nix and Gao 1998; Bull 2003; Kiener et al. 2009; Pharr et al. 2010). Elastic modulus, generally considered an intrinsic property of a monolithic material, typically does not exhibit a size effect (Kiener et al. 2009; Maier et al. 2011), but any occurrence of such an effect would suggest experimental artifacts such as surface roughness and substrate influence (Kiener et al. 2009; Zak et al. 2022). However, numerous studies indicate that shales as composite materials with complex microstructures do exhibit a certain size effect in elastic modulus (Wang et al. 2022; Yang et al. 2023). The slightly variable *E*_*r*_ for the tested single grains in this study may be attributed to grain surface roughness, i.e., a curtain-like structure (Figs. 3a, e, 5e), caused by the curtaining effect in BIB cross-sections (see also Desbois et al. 2011; Sondergeld et al. 2010).

The measurement became unstable and showed increasing damage when the loading rate was decreased to 1000 µN s^−1^ (Fig. 7), suggesting that the clay matrix may be sensitive to low indentation loading rates. It is worth noting that Maps 1–5 were indented with varied loading rates larger than 1000 µN s^−1^. The increasing surface damage observed at the low indentation loading rate is likely due to increasingly notable thermal drift of the system or a more pronounced influence of environmental vibrations when the indentation speed was low. Shi et al. (2019) tested nanoindentation loading rates of 5–30 mN s^−1^ and found that the contact hardness and Young’s modulus increase with increasing loading rate within an indentation depth range from 2 to 5 μm. In this study, within the tested depths up to 1.5 μm, no systematic change in mechanical properties with respect to the loading rate was observed. The loading rate is an extrinsic property compared to the other rate properties, such as strain-rate sensitivity, which is an intrinsic property (Maier et al. 2011; Wang et al. 2022). The relationship between the loading rate and the measured mechanical properties can be influenced by various factors, such as the magnitude of the loading rate, the indentation depth, the material itself, and the nanoindenter equipment. Based on the preliminary observations from this study, it is recommended to use high loading rates for experimental efficiency and stability, as well as to minimize viscous contributions. However, it should be noted that this recommendation is based on limited observations, and it is advisable to conduct a higher number of measurements using different loading rate settings to establish a more comprehensive understanding of the loading rate influence on micromechanical properties.

### Representativeness of clay matrix properties

The representativeness of the measured mechanical values for the clay matrix in the tested sample was assessed in terms of the representative area of the clay matrix and the number of data points needed to represent mechanical properties. The variation in the measured values (Table 2) was found to be associated with the inherent differences in the indented areas of the clay matrix, as observed in the SEM images of the nanoindentation imprints (Figs. 3, 5, 7, 9, 10). However, the representative mechanical values should be able to capture the intrinsic or inherent mechanical values for the clay matrix despite these variations.

**Fig. 13:**
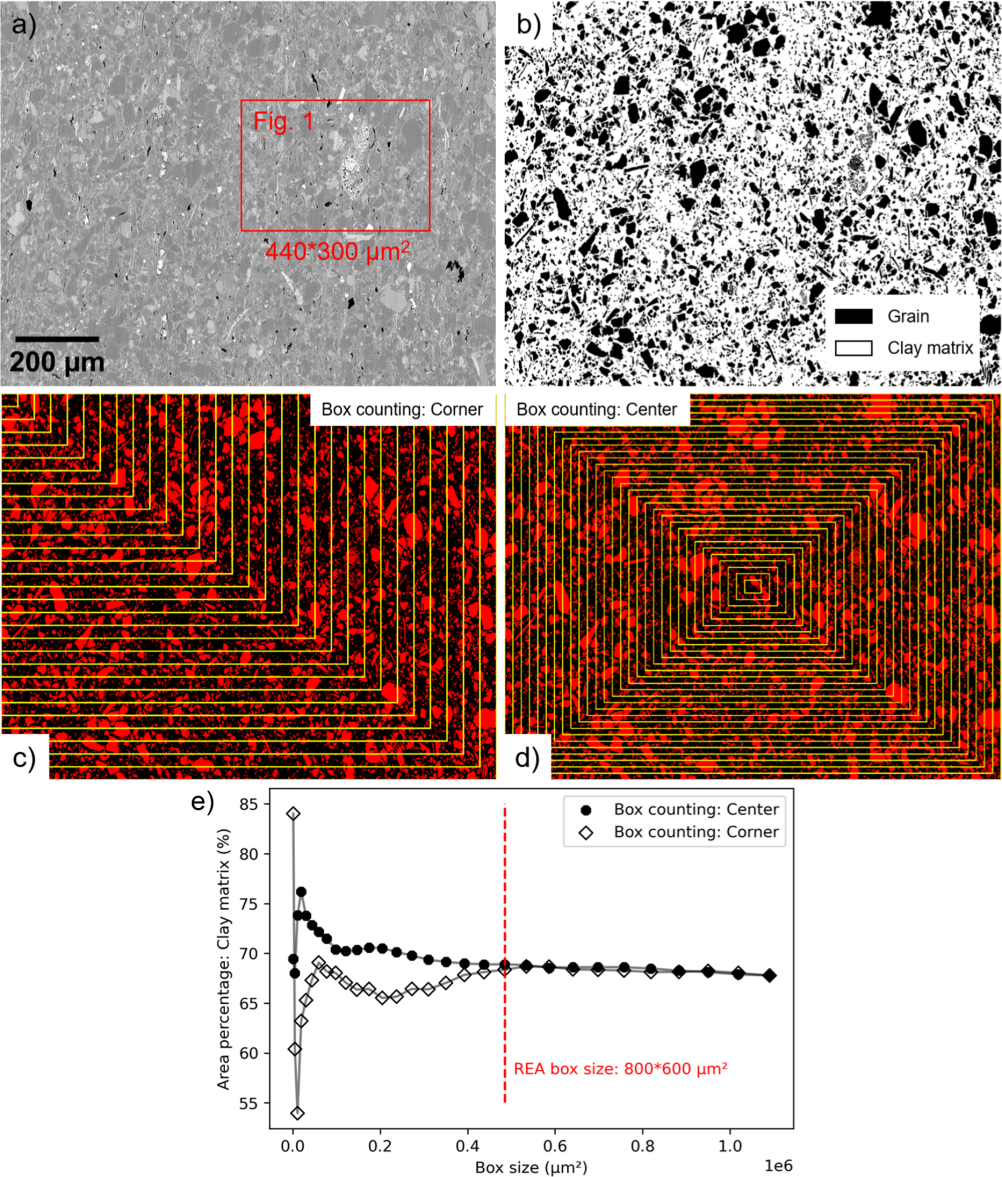
Determination of the representative elementary area for the studied sample. **a** SEM (BSE) image of the studied sample covering the tested area in Fig. 1. **b** Segmented binary image of the grain and clay matrix. **c**, **d** Box counting from the corner and the center in 30 steps. **e** Change of the clay matrix area percentage with the increasing box sizes indicated by yellow boxes in (**c**) and (**d**)

To determine the representative elementary area (REA) of the clay matrix, the box counting method was applied, which involves counting the amount of different phases in increasing box sizes until the contribution of each phase no longer significantly changes (see also Kameda et al. 2006; Klaver et al. 2012; Hemes et al. 2013; Houben et al. 2013; Misch et al. 2018a, b; Cosenza et al. 2019a, b). The SEM image of the tested sample was firstly segmented using ImageJ (Schindelin et al. 2015, 2012), after which the box counting method was applied with two starting points from the corner and center, respectively. This resulted in a REA of 800 × 600 µm^2^ (Fig. 13), which is significantly larger than reported in previous studies on shale samples, e.g., 140 × 140 μm^2^ by Klaver et al. (2012), 100 × 100 μm^2^ by Houben et al. (2013), 10,000–300,000 μm^2^ by Misch et al. (2018a, b). This difference may be due to the segmentation of compositional phases (i.e., mineral components) vs. the “structural-mechanical classes” used for classification in this study. The REA determined here is based on solely two phases, i.e., grain and clay matrix, and achieved with the stable value of ~ 68% of the clay matrix area for this specific sample. It is noted that the clay matrix in Fig. 13b contains clay aggregates and fine lithic fragments (< 3.9 µm), and large clay mineral platelets were classified as grain as they are stronger than “clay matrix” (Fig. 10). Based on the box counting analysis of this “structural-mechanical classes” segmentation, the area covered by the nanoindentation mapping shown in Fig. 1 was found to be smaller than the determined REA, indicating that it may not be representative in terms of covering the entire REA.

However, to cover the REA of 800 × 600 µm^2^ with an indentation spacing of 6 μm, approximately 13,333 indentations would be required. This is far beyond the number normally indented for shale or mudstone materials. Alternatively, the representativeness can be considered in terms of the number of data points needed to capture the mechanical properties. For single-phase materials like quartz, a few indentations would be sufficient to determine representative intrinsic mechanical values (e.g., Fig. 9b), although the spatial spreading of the quartz phase may be larger than that of a single quartz grain itself. Similarly, for the clay matrix, there may be a specific number of indentations needed to capture spatial variations in mechanical properties and achieve representativeness.

**Fig. 14:**
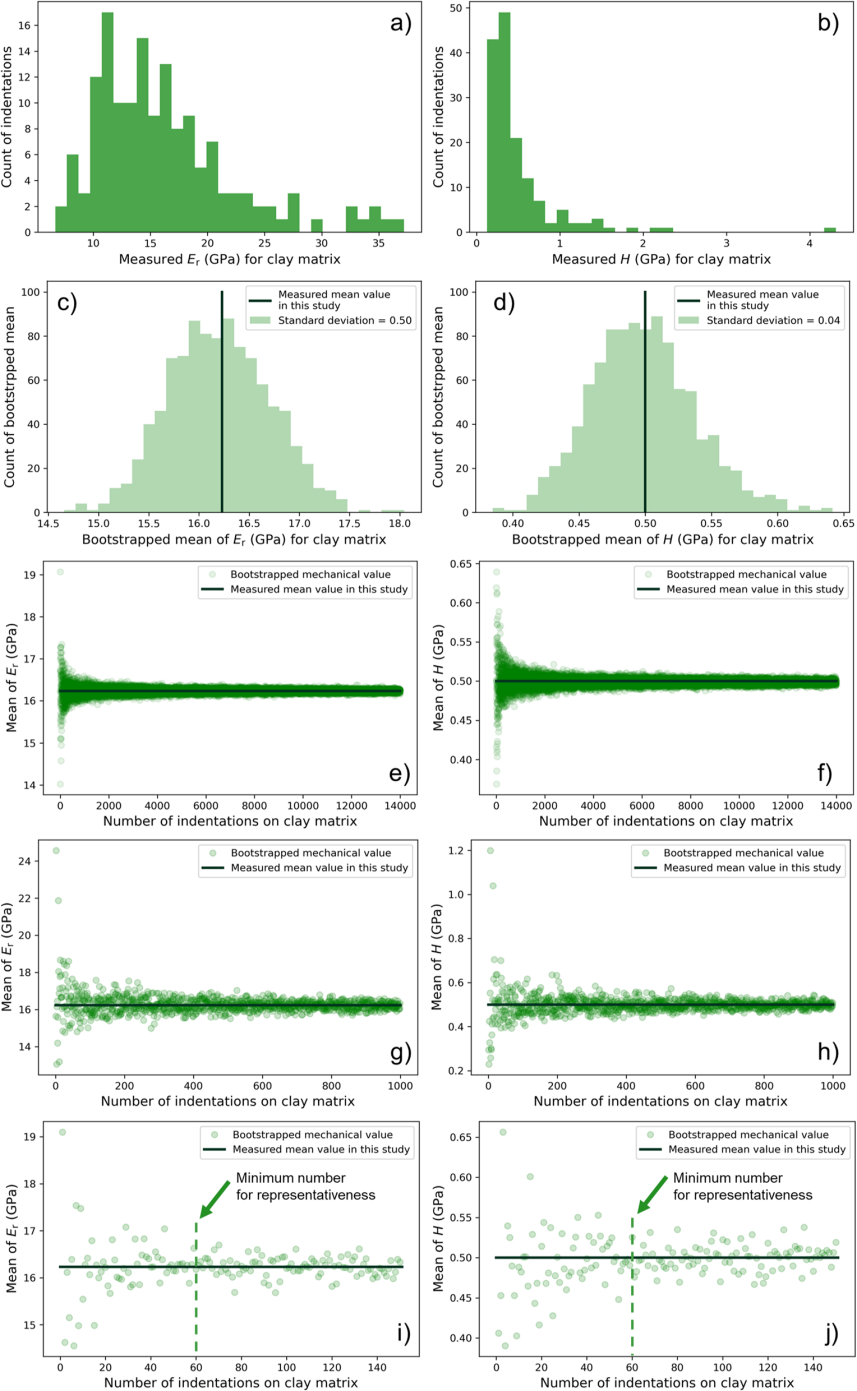
Bootstrapping analysis of the mechanical parameters of the clay matrix. **a**, **b** Histograms of *E*_*r*_ and *H* of the clay matrix. **c**, **d** Histograms of the bootstrapped means of *E*_*r*_ and *H* for the clay matrix. **e**, **f** Mean of *E*_*r*_ and *H* from bootstrapping with an increasing sample size to the maximum of 14,000 indentations. **g**, **h** Mean of *E*_*r*_ and *H* from bootstrapping with an increasing sample size to the maximum of 1000 indentations. **i**, **j** Mean of *E*_*r*_ and *H* from bootstrapping with an increasing sample size to the maximum of 151 indentations

To assess the representativeness of the measured cluster means, the bootstrapping method was applied. This method was initially introduced by Efron (1979) and is one of the most relevant advances in modern statistics. Bootstrapping was applied in multiple fields such as material science, earth science, environmental sciences, or biology (Henderson 2005; Roca-Pardiñas et al. 2009; Nordsvan et al. 2020; Becker et al. 2022). It involves resampling with replacement from the existing dataset (i.e., assumed representative sample from a larger population) to estimate statistical characteristics (Efron 1979). To create many simulated sub-samples from an original sample, two resampling scenarios were considered. In Scenario A the datasets were resampled 1000 times to the same size of the original dataset (n = 151 for clay matrix in Table 2), simulating 1000 repetitions of the nanoindentation mapping with 151 indents on the clay matrix. In this way, the consistency of the measured mean can be estimated. Scenario B involved resampling datasets with different sizes, ranging from 1 to the maximum size achieved (n = 14,000, 1000, or 151), resembling a repeated nanoindentation mapping with varying numbers of indents on the clay matrix. This allowed the determination of the minimum number of indents on the clay matrix needed to capture a consistent mean. The results of bootstrapping analysis are shown in Fig. 14. The original measured *E*_*r*_ and *H* for clay matrix in the histogram distributions are displayed for comparison (Fig. 14a, b). The histograms seem to be right-skewed but still resemble normal distributions, with the large upper range outliers included to capture the possible variability of the *k*-means clustering results. The histograms in Fig. 14c, d show the distribution of 1000 means calculated from 1000 times resampling in Scenario A, following the normal distributions. The measured cluster mean values align well with the means of the normal distribution, indicating good representativeness. The standard deviation of these bootstrapped means represents the standard error of the measured mean: 16.23 ± 0.5 GPa for *E*_*r*_ and 0.50 ± 0.04 GPa for* H*, which provides an uncertainty estimation for the measured mean. The lower standard error values suggest the measured means represent the base population very well. The results from Scenario B show that a stable mean close to the measured cluster mean was quickly achieved with ~ 60 indentation data points (Fig. 14e–j). This number is smaller than the total number of indentation (n = 151), indicating that a statistically representative mean can be obtained with a relatively small number of data points. Nevertheless, it is still recommended to distribute the mapping area more widely to capture possible larger-scale heterogeneities in the spatial distribution of mineral constituents and to ideally cover the entire REA. A promising approach planned for the future is to use cutting-edge devices such as FemtoTools FT-NMT04, known for its fast and accurate acquisition of high-resolution nanoindentation maps (e.g., Dhal et al. (2022) tested the mapping of 40,000 indents covering an area of 150 × 150 µm^2^ on alloys).

Overall, while it is an experimental challenge to cover the representative area of the clay matrix in the tested sample by a nanoindentation mapping array, a smaller number of data points is actually required to achieve stable and representative mechanical values.

## Conclusion

In this study, a novel workflow combining high-speed nanoindentation mapping and machine learning data analysis was developed to characterize the micromechanical properties of the clay matrix in mudstones. The application of *k*-means clustering successfully distinguished the mechanical characteristics of the clay matrix, brittle mineral grains, and a transitional class including areas affected by grain boundary effects or artefacts such as cracks. The resulting average *E*_r_ and *H* values of the clay matrix in the studied mudstone sample range at 16.2 ± 6.2 GPa and 0.5 ± 0.5 GPa, respectively. The clustering results were consistent with observations from SEM images, despite differences in nanoindentation test settings and indenter tips, underlining a robust assessment scheme.

The sensitivity of indentation measurements to various factors such as indentation depth, indenter tip, and loading rate was investigated. The clay matrix exhibited relatively consistent mechanical properties across a wide range of contact depths (~ 150–1750 nm) for the Berkovich and Cube Corner tips, indicating limited sensitivity of mechanical properties to indentation depth and tip geometry. However, the individual measurements tended to become unstable at a lower loading rate (1000 μN s^−1^), suggesting that the clay matrix may be sensitive to such conditions due to viscous contributions.

To assess the representativeness of the clay matrix, the box counting and bootstrapping methods were employed. The results revealed experimental challenges in covering the representative area of the clay matrix for the tested sample (REA of 800 × 600 µm^2^). However, achieving representativeness in terms of the number of indentations required much fewer data points (n = 60), which corresponds to a considerably smaller area compared to the determined REA.

In summary, this study presents a significant methodological advancement in the characterization of mudstones and similar fine-grained sedimentary rocks. The application of machine learning-based clustering combined with high-speed nanoindentation mapping offers an efficient and promising tool for future studies on the micromechanical properties of mudstones.

### Supplementary Information

Below is the link to the electronic supplementary material.

Supplementary file1 (DOCX 2115 KB)

## Data Availability

Data will be made available on request. No datasets were generated or analysed during the current study.
