# Supplementary material for: Mapping the composite nature of clay matrix in mudstones: integrated micromechanics profiling by high-throughput nanoindentation and data analysis
Source: Geomech Geophys Geo Energy Ge Resour. 2024 Aug 14;10(1):139. doi: 10.1007/s40948-024-00864-9 (PMC11322212; doi:10.1007/s40948-024-00864-9)
Supplement: Supplementary file 1 — Supplementary file1 (DOCX 2115 KB) [file 40948_2024_864_MOESM1_ESM.docx]

# Supplementary data


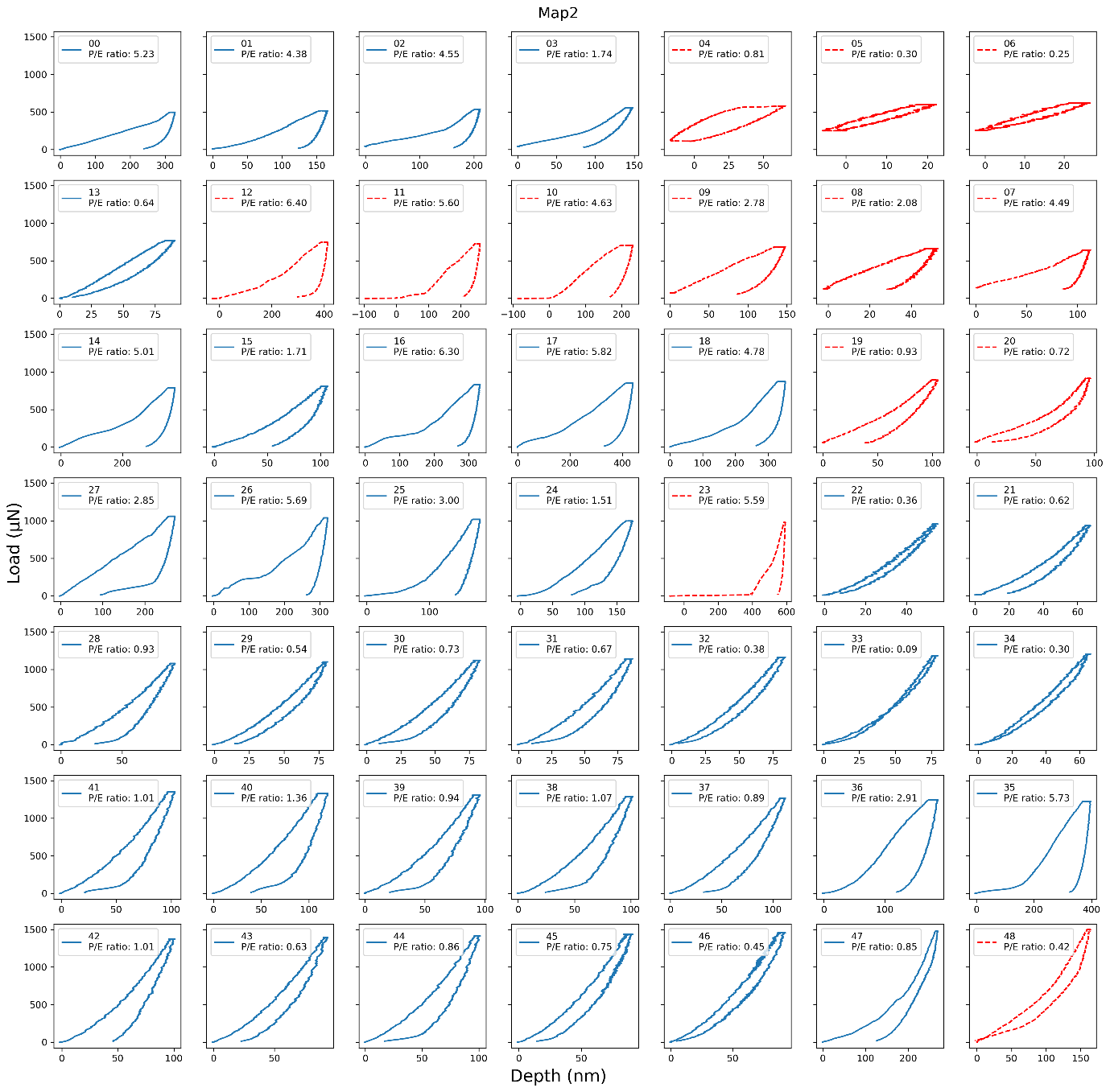
 A1. Quality control for load-displacement curves of Map2. Invalid curves are marked in red dash lines after the quality control (e.g., loads and displacements do not start at the zero). P/E ratio—ratio of plastic to elastic contributions.


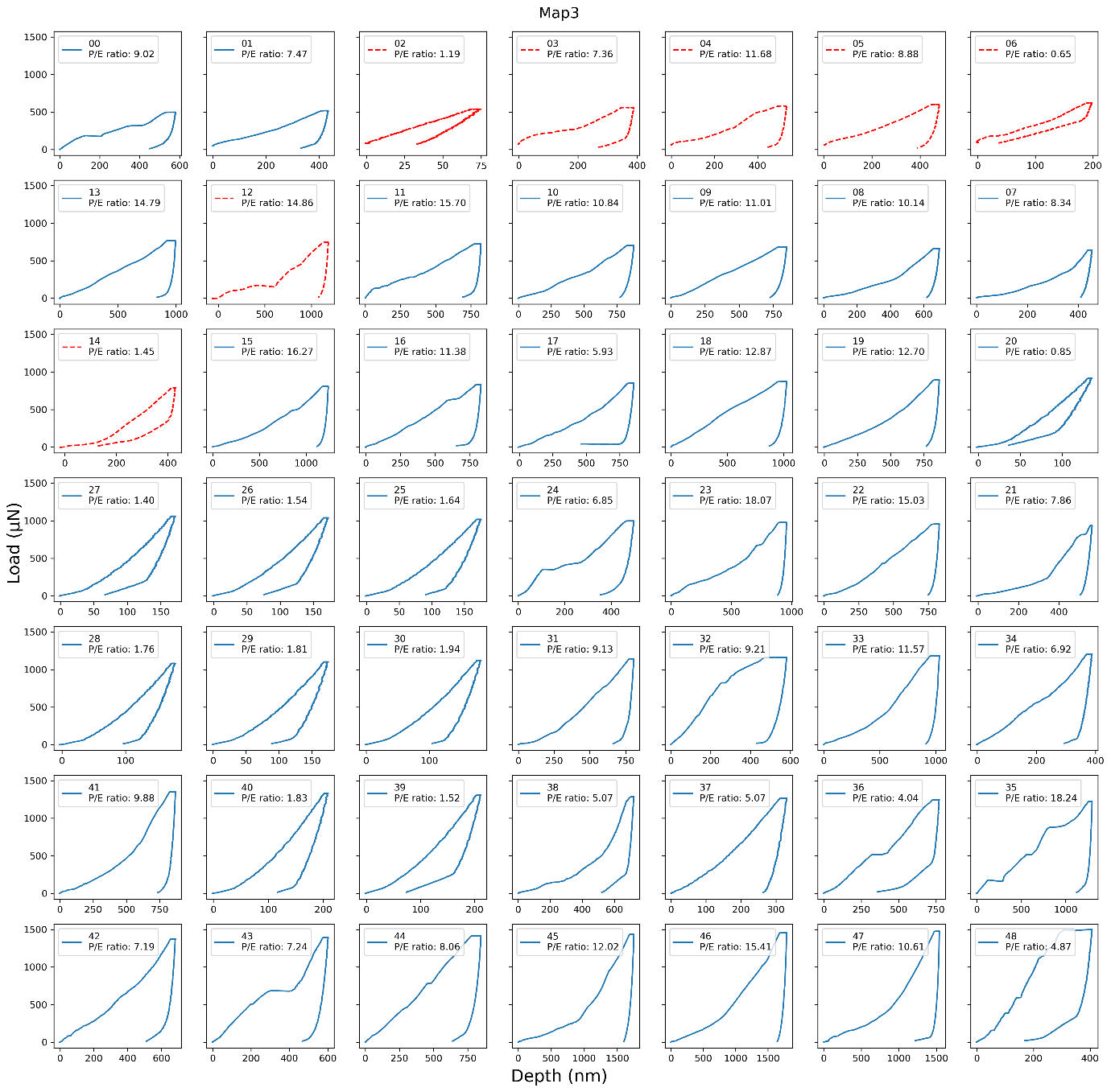
 A2. Quality control for load-displacement curves of Map3. Invalid curves are marked in red dash lines after the quality control (e.g., loads and displacements do not start at the zero). P/E ratio—ratio of plastic to elastic contributions.


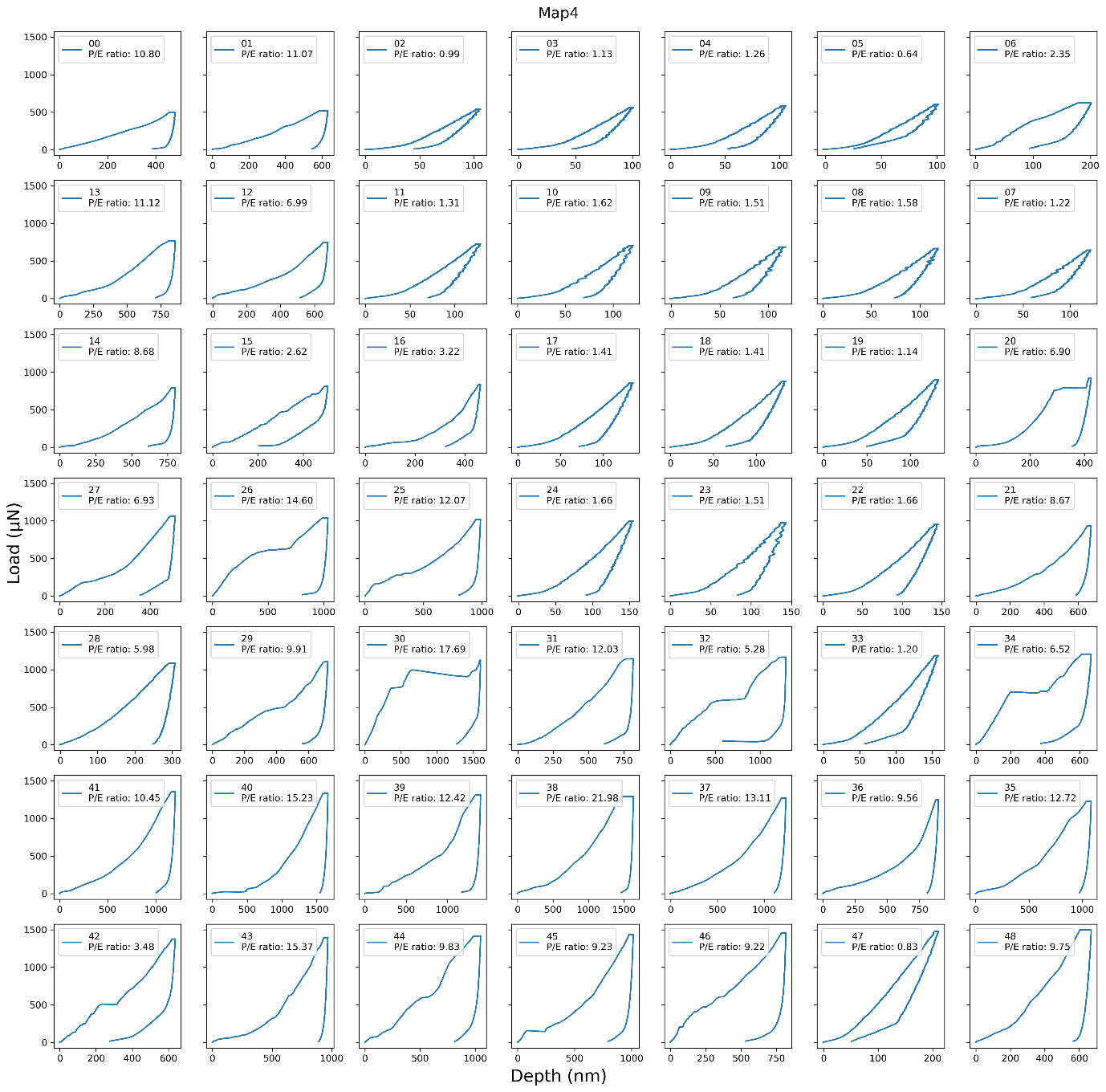
 A3. Quality control for load-displacement curves of Map4. Invalid curves are marked in red dash lines after the quality control (e.g., loads and displacements do not start at the zero). P/E ratio—ratio of plastic to elastic contributions.


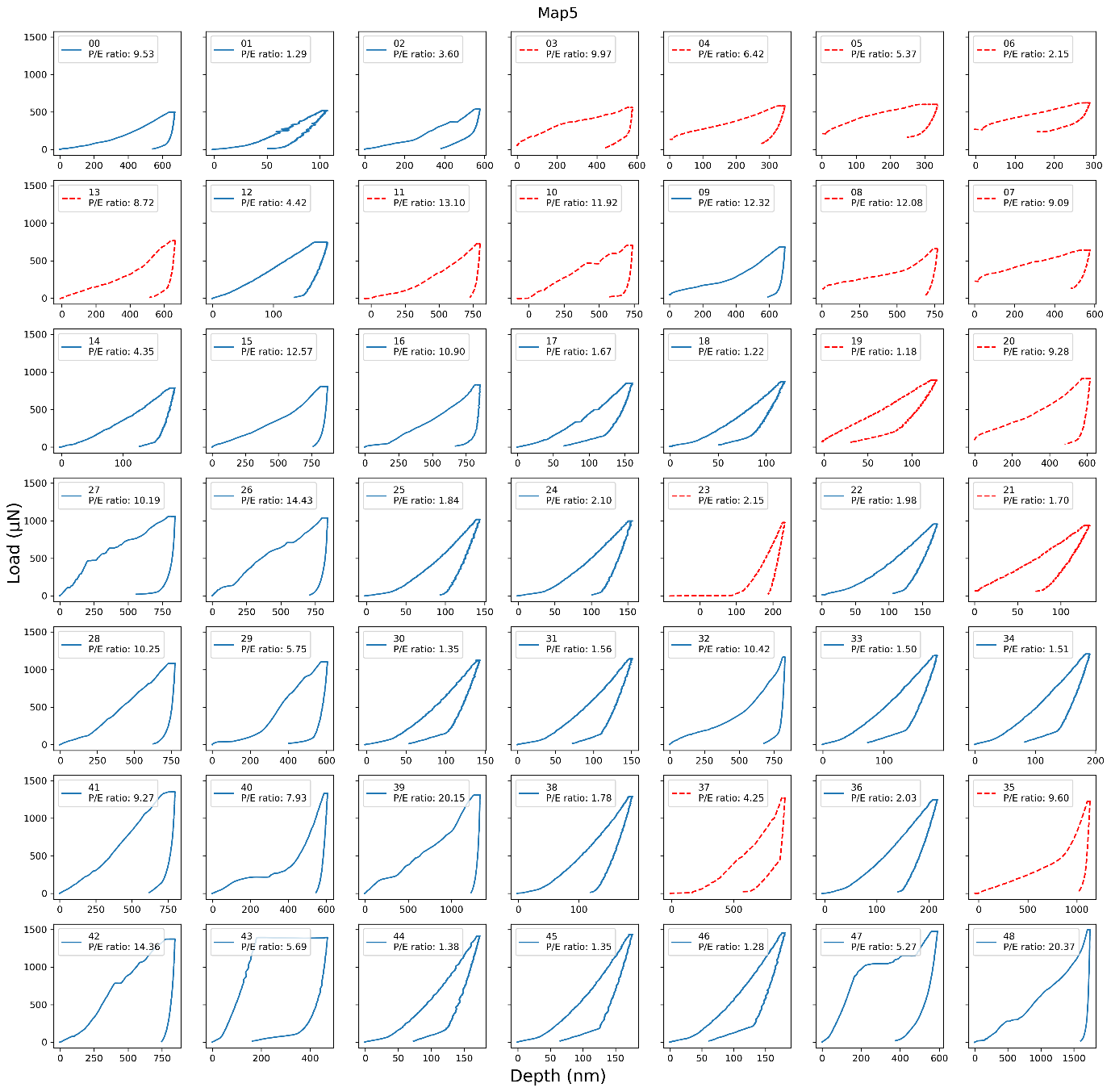
 A4. Quality control for load-displacement curves of Map5. Invalid curves are marked in red dash lines after the quality control (e.g., loads and displacements do not start at the zero). P/E ratio—ratio of plastic to elastic contributions.


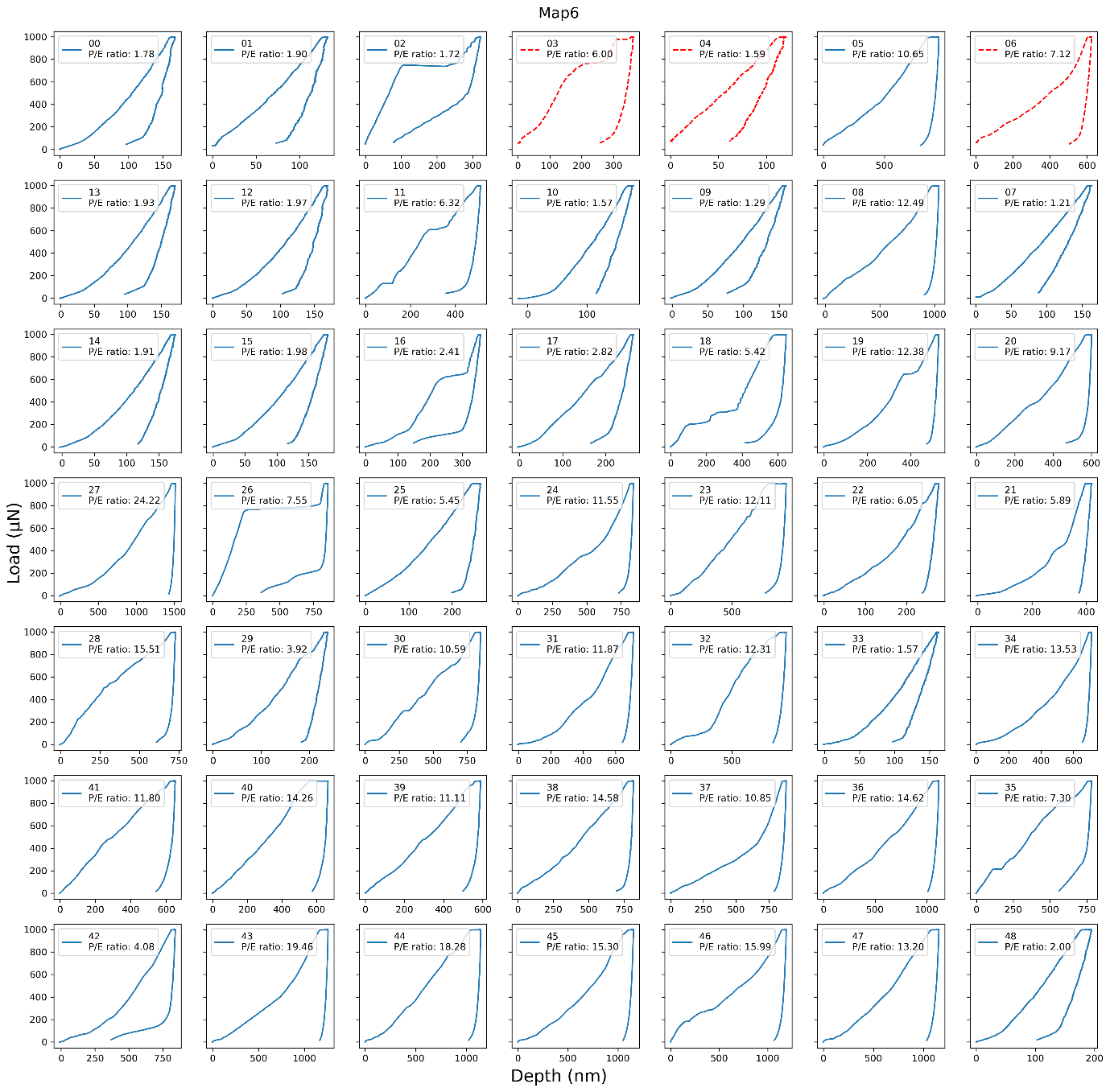
 A5. Quality control for load-displacement curves of Map6. Invalid curves are marked in red dash lines after the quality control (e.g., loads and displacements do not start at the zero). P/E ratio—ratio of plastic to elastic contributions.


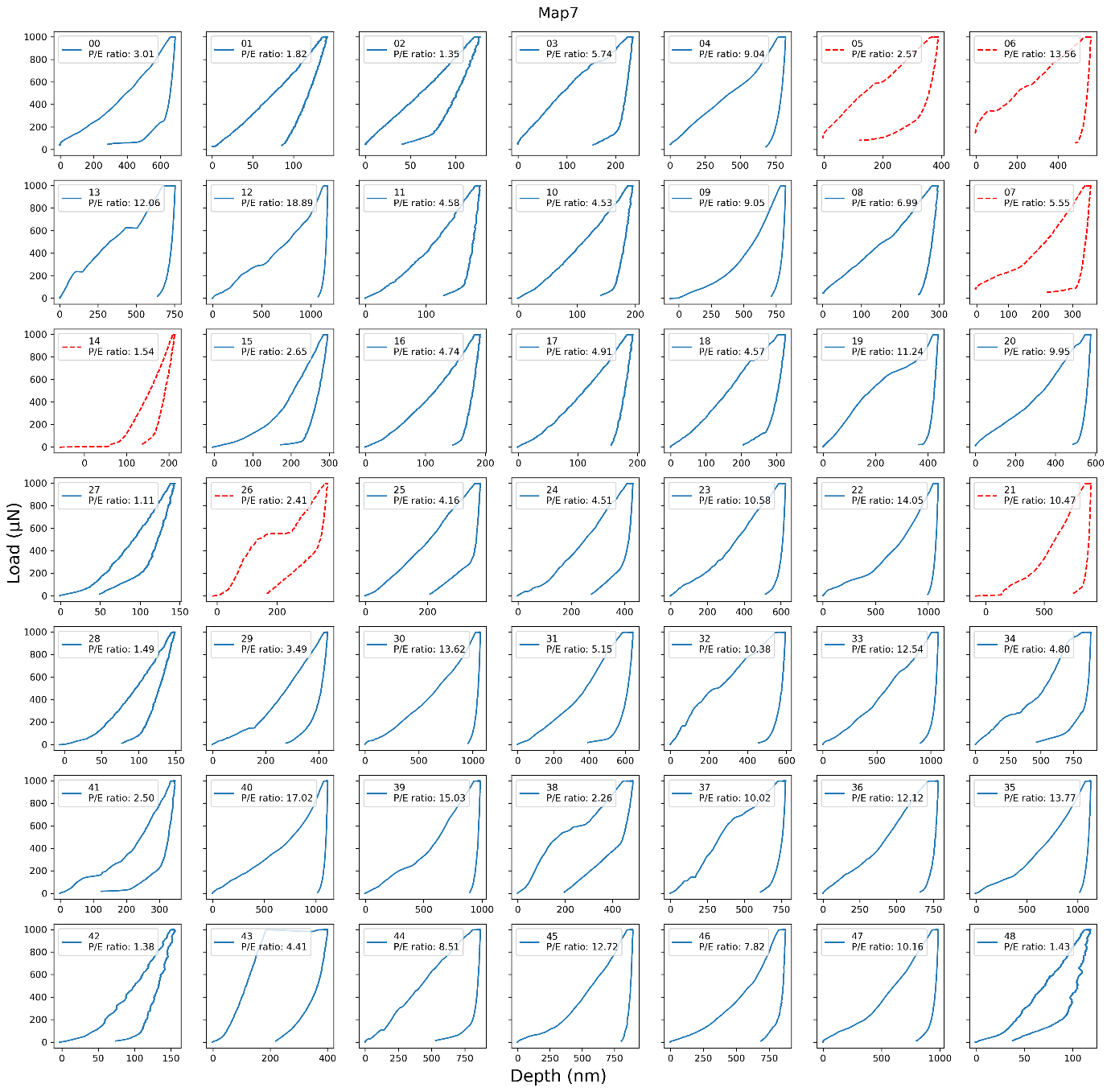
 A6. Quality control for load-displacement curves of Map7. Invalid curves are marked in red dash lines after the quality control (e.g., loads and displacements do not start at the zero). P/E ratio—ratio of plastic to elastic contributions.


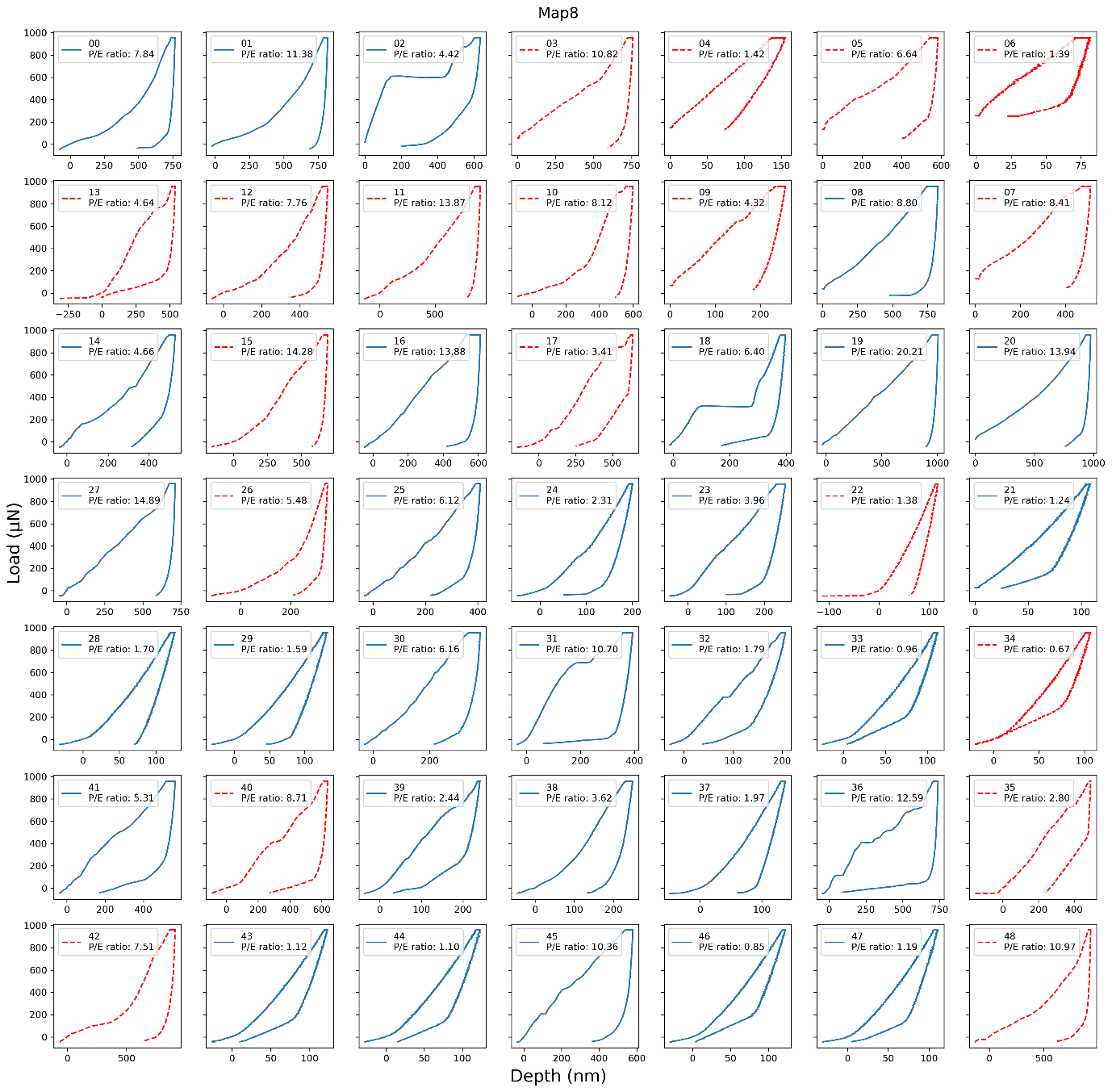
 A7. Quality control for load-displacement curves of Map8. Invalid curves are marked in red dash lines after the quality control (e.g., loads and displacements do not start at the zero). P/E ratio—ratio of plastic to elastic contributions.
